# Targeting DPP9 attenuates podocyte injury by regulating NRF2 antioxidant signaling

**DOI:** 10.3389/fphar.2026.1930044

**Published:** 2026-09-17

**Authors:** Chenkai Cui, Shizhuo Wei, Jianpeng Zhang, Jing Xu, Shokhida Naimova, Yan Li, Haodong Wang, Yinhong Wang, Ruoyan Si, Linting Wei, Fuqian Lei, Xiaoyong Yu, Pengfei Liu, Rongguo Fu

**Affiliations:** 1 Department of Nephrology, Second Affiliated Hospital of Xi’an Jiaotong University, Xi’an, China; 2 International Joint Research Center on Injury and Repair of Urinary System, Shaanxi International Science and Technology Cooperation Base, Second Affiliated Hospital of Xi’an Jiaotong University, Xi’an, China; 3 Department of Nephrology, Bukhara State Medical Institute, Bukhara, Uzbekistan; 4 Department of Ophthalmology, Second Affiliated Hospital of Xi’an Jiaotong University, Xi’an, China; 5 Department of Nephrology, Shaanxi Traditional Chinese Medicine Hospital, Xi’an, China; 6 International Joint Research Center on Cell Stress and Disease Diagnosis and Therapy, National and Local Joint Engineering Research Center of Biodiagnosis and Biotherapy, Second Affiliated Hospital of Xi’an Jiaotong University, Xi’an, China

**Keywords:** CKD, DPP9, finerenone, NRF2, oxidative stress, podocyte injury

## Abstract

**Introduction:**

Podocyte injury is a critical event in the progression of chronic kidney disease (CKD), with oxidative stress as a central pathogenic mechanism. Despite the efficacy of finerenone in mitigating CKD progression, its precise cytoprotective mechanisms within podocytes remain incompletely defined. This study aimed to elucidate the role of dipeptidyl peptidase 9 (DPP9) in podocyte injury and determine whether finerenone exerts its renoprotective effects via DPP9‐mediated antioxidant effects.

**Methods:**

Integrated bioinformatic analyses of single‐cell and bulk transcriptomic databases (KIT, NephroSeq) were performed, with validation in renal biopsies from patients with diabetic kidney disease (DKD) and in murine models of DKD and adriamycin‐induced nephropathy. *In vitro*, immortalized human podocyte (HPC) cells were stimulated with high glucose or adriamycin, with DPP9 knockdown or overexpression. RNA sequencing and immunoprecipitation assays were utilized to explore the underlying molecular mechanisms and protein interactions.

**Results:**

DPP9 was significantly downregulated in injured podocytes from both human and murine models. DPP9 overexpression activated NRF2‐mediated antioxidant responses, protecting podocytes from injury, whereas DPP9 depletion exacerbated cellular damage. Finerenone upregulated DPP9 protein levels, which subsequently enhanced DPP9-KEAP1 binding and competitively disrupted the NRF2‐KEAP1 interaction, leading to NRF2 stabilization and activation. Consequently, finerenone improved renal function and alleviated pathological damage *in vivo*.

**Conclusion:**

This study identifies DPP9 as a critical mediator of podocyte antioxidant defense and establishes that the therapeutic efficacy of finerenone is, at least in part, dependent on the upregulation of DPP9.

## 1 Introduction

Chronic kidney disease (CKD) and its progression to end-stage renal disease (ESRD) represent a global public health problem. Currently, the global prevalence of CKD is estimated to be approximately 850 million people, with about four million individuals suffering from ESRD (Herrington et al., 2026). Among the numerous causes of CKD and ESRD, glomerular diseases occupy a central position (Cunanan et al., 2025). Podocytes are essential components of the glomerular filtration barrier (GFB), as their structural integrity and functional stability are pivotal for maintaining normal renal filtration (Kopp et al., 2020; Shankland, 2006). Podocyte injury directly disrupts GFB integrity in various primary and secondary glomerular diseases (Sever and Schiffer, 2018), and it is the initiating and core event for proteinuria and progressive loss of renal function (Qi et al., 2017; Putra et al., 2023). However, current therapeutic options for podocyte injury remain limited. Therefore, elucidating the key pathological mechanisms of progressive glomerular diseases is crucial for developing effective interventions, and targeted podocyte protection has emerged as a core strategy to prevent the progression of CKD to ESRD.

Dipeptidyl peptidase 9 (DPP9), a member of the serine protease family, regulates a variety of biological processes through its enzymatic activity and non-enzymatic functions (Zhang et al., 2013; Cui et al., 2022). We first reported that DPP9 is expressed in renal proximal tubule epithelial cells, and promotes epithelial-mesenchymal transition in tubulointerstitial fibrosis (Zhang et al., 2021). Recent studies have shown that DPP9 competitively binds KEAP1 to modulate NRF2 and ROS levels in clear cell renal cell carcinoma and hepatocellular carcinoma cells (Chang et al., 2023; Zhou et al., 2024). By reviewing DPP9-stained biopsy slides, we also observed DPP9 was expressed in podocytes. However, its function in podocytes remains unknown.

Oxidative stress is a common mediator of podocyte injury in glomerular diseases (Ilatovskaya et al., 2025). In the hyperglycemic microenvironment of diabetic kidney disease (DKD), or upon stimulation by advanced glycation end products (AGEs), the redox balance is disrupted in podocytes, leading to excessive reactive oxygen species (ROS) accumulation and subsequent oxidative stress (Li et al., 2023; Efiong et al., 2024). Nuclear factor erythroid 2-related factor 2 (NRF2) is a master transcription factor that regulates cellular antioxidant defense. It binds to antioxidant response elements (AREs) in the promoter regions of target genes, activating the transcription of antioxidant enzymes (e.g., heme oxygenase-1 [HMOX1], glutamate-cysteine ligase modifier subunit [GCLM], aldo-keto reductase family 1 member C1 [AKR1C1]) that scavenge excess ROS (Nguyen et al., 2003; Shi et al., 2023). Under basal conditions, Kelch-like ECH-associated protein 1 (KEAP1) mediates the ubiquitination and proteasomal degradation of NRF2, maintaining cytoplasmic NRF2 levels (Cullinan et al., 2004; Liu et al., 2019). Notably, NRF2 knockdown in human podocytes amplifies the downregulation of podocyte marker proteins under high-glucose conditions (Zhang et al., 2018), confirming that NRF2 dysfunction contributes to podocyte vulnerability to oxidative stress in DKD. This raises the question of whether DPP9 acts as a regulator of oxidative stress in podocytes. Here, we found that DPP9 orchestrates oxidative stress responses within podocytes via NRF2. Furthermore, we also found that finerenone, a novel non-steroidal mineralocorticoid receptor antagonist (MRA), could upregulate DPP9 and NRF2 in podocytes.

In recent clinical trials, finerenone has demonstrated clinical efficacy in slowing CKD progression and reducing proteinuria (Alhomoud et al., 2025; Agarwal et al., 2025). Notably, mineralocorticoid receptor (MR) is not only expressed in renal tubules but also in podocytes (Nakamura et al., 2022), suggesting that finerenone may exert direct protective effects on podocytes. However, the molecular mechanisms underlying finerenone’s podocyte-protective effects remain elusive. Given that MR activation promotes ROS production and leads to oxidative damage (Nakamura et al., 2022; Barrera-Chimal et al., 2022; Jaisser and Barrera-Chimal, 2025), and considering the key role of oxidative stress in podocyte injury, we postulated that finerenone exerts its renoprotective effects, at least in part, through the regulation of oxidative stress in injured podocytes.

This study found that DPP9 was significantly downregulated in injured podocytes from patients with DKD and in murine models. DPP9 overexpression activated NRF2-mediated antioxidant responses and protected podocytes against high glucose (HG)- or adriamycin (ADR)-induced injury, whereas DPP9 depletion exacerbated podocyte damage. Importantly, finerenone upregulated DPP9, improved renal function and attenuated pathological damage in ADR-induced podocyte injury model. Finerenone upregulated DPP9 protein levels, which subsequently enhanced DPP9–KEAP1 binding and competitively disrupted the NRF2–KEAP1 interaction. This led to NRF2 stabilization and activation, ultimately preserving podocyte cytoskeletal proteins and ameliorating podocyte injury.

## 2 Materials and methods

### 2.1 Reagents

All reagents and antibodies were listed in Supplementary Tables S1, S2.

### 2.2 Human renal biopsy samples

Human renal biopsy samples were obtained from the Second Affiliated Hospital of Xi’an Jiaotong University, including renal biopsies from 5 DKD patients and normal tumor-adjacent kidney tissue from five renal carcinoma patients without diabetes or kidney disease history. All procedures were approved by the Biomedical Ethics Committee of Health Science Center of Xi’an Jiaotong University (Institutional Review Board approval number: 2021-839).

### 2.3 Cell culture, viral infection, and plasmid transfection

The conditionally immortalized human podocyte cell line (HPC) was gifted by Prof. Fan Yi (Department of Pharmacology, Shandong University, China). HPC cells were proliferated at 33 °C in RPMI 1640 medium supplemented with 10% fetal bovine serum, 100 U/mL penicillin, 0.1 mg/mL streptomycin, and 1× insulin-transferrin-selenium (ITS). Upon reaching the required cell number, cells were transferred to 37 °C in ITS-free medium for differentiation, which was maintained for 2 weeks before subsequent experiments.

HPC cells were infected with lentivirus carrying DPP9 shRNA or KEAP1 shRNA (with GFP and puromycin resistance genes). After 2 days, cells were cultured in medium containing 2.0 μg/mL puromycin to select stable DPP9-knockdown cell lines and KEAP1-knockdown cell lines. DPP9 and KEAP1 knockdown efficiency was confirmed by Western blot (WB). Plasmids containing full-length human DPP9 cDNA, Flag-tagged NRF2, and HA-tagged ubiquitin (Ub) were transiently transfected into HPC cells using Lipofectamine 3000 transfection reagent according to the manufacturer’s instructions. The plasmid-transfection reagent complexes were removed after 4 h of incubation. Cells were further cultured for 24 h, then harvested for WB validation.

### 2.4 Animal experiments

Animal studies were approved by the Biomedical Ethics Committee of Health Science Center of Xi’an Jiaotong University (Ethics approval number: XJTUAE2025-2229; Approval date: 5 March 2025).

#### 2.4.1 Diabetic kidney disease (DKD) mice model

Male C57BL/6 mice (6-week-old) were fed a high-fat diet (HFD) for 4 weeks, followed by unilateral nephrectomy. After 1 week, diabetes was induced by intraperitoneal streptozotocin (STZ; 50 mg/kg in 50 mmol/L citrate buffer, pH 4.5) for three consecutive days, and mice were then maintained on HFD. One week after the final STZ injection, diabetes was confirmed by blood glucose >16.7 mmol/L. Urinary albumin and glucose were monitored weekly. Mice were sacrificed 12 weeks later, and blood and renal tissues were collected for subsequent experiments.

#### 2.4.2 Adriamycin-induced nephropathy (AN) mice model

Male Balb/c mice (10-week-old) were randomly divided into four groups: control group, finerenone group, AN group and AN + finerenone group. Mice in the AN groups received a single tail vein injection of adriamycin (12 mg/kg) to establish the AN model. Finerenone (10 mg/kg/day, oral gavage) (Luettges et al., 2022) was administered starting from 1 day after ADR injection. Urinary albumin was monitored weekly. Mice were sacrificed 8 weeks after adriamycin injection. Blood and renal tissues were collected for subsequent experiments.

### 2.5 Histopathology and scoring

Mouse renal tissues were fixed in 4% paraformaldehyde (PFA), embedded in paraffin, and sectioned at 4 μm for hematoxylin-eosin (H&E) staining, Masson staining, and periodic acid-Schiff (PAS) staining. Semi-quantitative scoring of glomerulosclerosis in PAS-stained sections was performed as follows: 0, no sclerosis; 1, sclerosis in <10% of the glomerulus; 2, sclerosis in 10%–25% of the glomerulus; 3, sclerosis in 25%–50% of the glomerulus; 4, sclerosis in >50% of the glomerulus. Fifty glomeruli per sample were scored, and the mean value was calculated. Semi-quantitative scoring of renal interstitial fibrosis in Masson-stained sections was performed as follows: 0, no fibrosis; 1, fibrosis in <10% of the area; 2, fibrosis in 10%–25% of the area; 3, fibrosis in 25%–50% of the area; 4, fibrosis in >50% of the area. Twenty fields per sample were scored, and the mean value was calculated (Tian et al., 2014).

### 2.6 Biochemical assays

Urine albumin levels were detected using an albumin ELISA kit. Plasma and urine creatinine levels were measured using a creatinine assay kit. Plasma urea nitrogen levels were determined using a urea nitrogen assay kit, all according to the manufacturers’ protocols.

### 2.7 Immunofluorescence

#### 2.7.1 Cellular immunofluorescence

Cells were seeded on cell climbing slides. At room temperature, cells were fixed with 4% PFA (15 min), permeabilized with 0.3% Triton X-100 (10 min), and blocked with 10% goat serum at 37 °C (1 h). Cells were incubated with primary antibodies at 4 °C overnight, followed by incubation with corresponding fluorescent secondary antibodies at room temperature for 1 h the next day. Nuclei were stained with DAPI. Images were captured using a fluorescence microscope and analyzed using ImageJ.

#### 2.7.2 Tissue immunofluorescence

Paraffin-embedded renal tissue sections (4 μm) were dewaxed and rehydrated. After antigen retrieval, sections were blocked with 10% goat serum at 37 °C for 1 h, then incubated with primary antibodies at 4 °C overnight. The next day, sections were incubated with corresponding fluorescent secondary antibodies at room temperature for 1 h. Nuclei were stained with DAPI. Images were captured using a fluorescence microscope and analyzed using ImageJ.

### 2.8 Immunohistochemistry (IHC)

Paraffin-embedded renal tissue sections (4 μm) were dewaxed and rehydrated. After antigen retrieval, sections were incubated with 3% H_2_O_2_ at room temperature for 10 min to block endogenous peroxidase activity, then blocked with 10% goat serum at 37 °C for 1 h. Sections were incubated with primary antibodies at 4 °C overnight, followed by incubation with HRP-conjugated secondary antibodies at room temperature for 1 h the next day. Staining was performed using DAB reagent, and sections were counterstained with hematoxylin. Images were captured using a microscope.

### 2.9 Western blot (WB)

Proteins from tissues and cells were extracted using RIPA lysis buffer containing protease inhibitors and phosphatase inhibitors. Protein concentrations were determined using a BCA protein assay kit. Proteins were separated by 7.5% or 10% SDS-PAGE and transferred to PVDF membranes. Membranes were blocked with 5% non-fat milk at room temperature for 1–2 h, then incubated with primary antibodies at 4 °C overnight. After washing, membranes were incubated with corresponding HRP-conjugated IgG secondary antibodies at room temperature for 1 h. Bands were visualized using an enhanced chemiluminescence kit.

### 2.10 Quantitative real-time PCR (qRT-PCR)

Total RNA was extracted using TRIzol reagent. RNA concentration was measured using a NanoDrop 2000 spectrophotometer (Thermo). RNA was reverse-transcribed into cDNA using PrimeScript RT Master Mix. Real-time qPCR was performed using TB Green Premix Ex Taq with specific primers. Primer sequences were listed in Supplementary Table S3.

### 2.11 Cell viability assay

HPCs were seeded in 96-well plates. After treatment, 10 μL of CCK-8 solution was added to each well, and the plate was incubated at 37 °C for 2 h. Absorbance was measured at 450 nm using a microplate reader.

### 2.12 ROS detection

Intracellular ROS levels were detected using an ROS assay kit. HPCs were seeded in 6-well plates. After treatment, cells were incubated with 10 μM 2′, 7′-dichlorodihydrofluorescein diacetate (DCFH-DA) at 37 °C for 20 min. Residual DCFH-DA was removed by washing. Images were captured and analyzed using a fluorescence microscope.

### 2.13 RNA sequencing

Total RNA was extracted from finerenone-treated and control HPC cells using TRIzol reagent. RNA sequencing libraries were constructed using the Illumina Stranded mRNA Prep Ligation Kit (Illumina, San Diego, CA, United States) according to the manufacturer’s instructions. Paired-end sequencing (2 × 150 bp read length) was performed on the Illumina NovaSeq X Plus platform (Illumina) at Shanghai Majorbio Biopharm Technology Co., Ltd. (Shanghai, China).

### 2.14 Immunoprecipitation (IP)

Cellular proteins were extracted using Western and IP lysis buffer containing protease inhibitors and phosphatase inhibitors. The supernatant of cell lysates was incubated with KEAP1 antibody or isotype control antibody at 4 °C overnight on a rotator, followed by the addition of Protein A + G Agarose and incubation at 4 °C for 2 h on a rotator. Precipitates were washed three times with pre-cooled IP buffer, then eluted by boiling in loading buffer for WB.

The ubiquitination assay was performed in Flag-tagged NRF2 and HA-tagged Ub transfected HPC cells. The supernatant of cell lysates was incubated with anti-Flag affinity gel at 4 °C overnight on a rotator, and ubiquitination of NRF2 was evaluated using WB.

### 2.15 Statistical analysis

All data are presented as the mean ± standard deviation (SD). Statistical analyses were performed using SPSS 27.0 statistical software (IBM Corporation, Armonk, NY, United States) and GraphPad Prism 10.0 (GraphPad Software, Boston, MA, United States). For comparisons between two groups, Student’s two-tailed unpaired *t*-test was used. For comparisons among multiple groups, one-way ANOVA followed by Tukey’s *post hoc* test was performed.

## 3 Results

### 3.1 Downregulated DPP9 expression in injured podocytes

*DPP9* expression was decreased in DKD patients’ podocytes compared with control patients from the Kidney Interactive Transcriptomics (KIT) database (Kidney Interactive Transcriptomics) (Figures 1A,B). The downregulated *Dpp9* was also found in glomeruli of male diabetic mice based on data from the Nephroseq transcriptome database (V5) (Supplementary Figure S1A) (Nephroseq transcriptome database v5). To determine the localization of DPP9 in podocytes, DPP9 and the podocyte cytoskeletal protein Synaptopodin were stained by immunofluorescence of human renal biopsies and mouse kidney. DPP9 was co-localized in Synaptopodin-positive podocytes (Figures 1C,I,K). Consistent with the transcriptional data, DPP9 protein levels were significantly reduced in glomeruli of DKD patients compared to normal tumor-adjacent kidney tissue shown by immunofluorescence staining (Figure 1D). Furthermore, DPP9 protein levels in the renal cortex were decreased in both DKD and adriamycin-induced nephropathy (AN) mouse models (Figures 1E–H). A significant reduction of glomerular DPP9 in both DKD and AN mice was further confirmed by IHC (Supplementary Figures S1B–E). Moreover, both the fluorescence intensity of DPP9 and its co-localization with Synaptopodin were significantly decreased in the glomeruli of DKD and AN mice compared with controls (Figures 1I–L), which is consistent with the human data.

**FIGURE 1:**
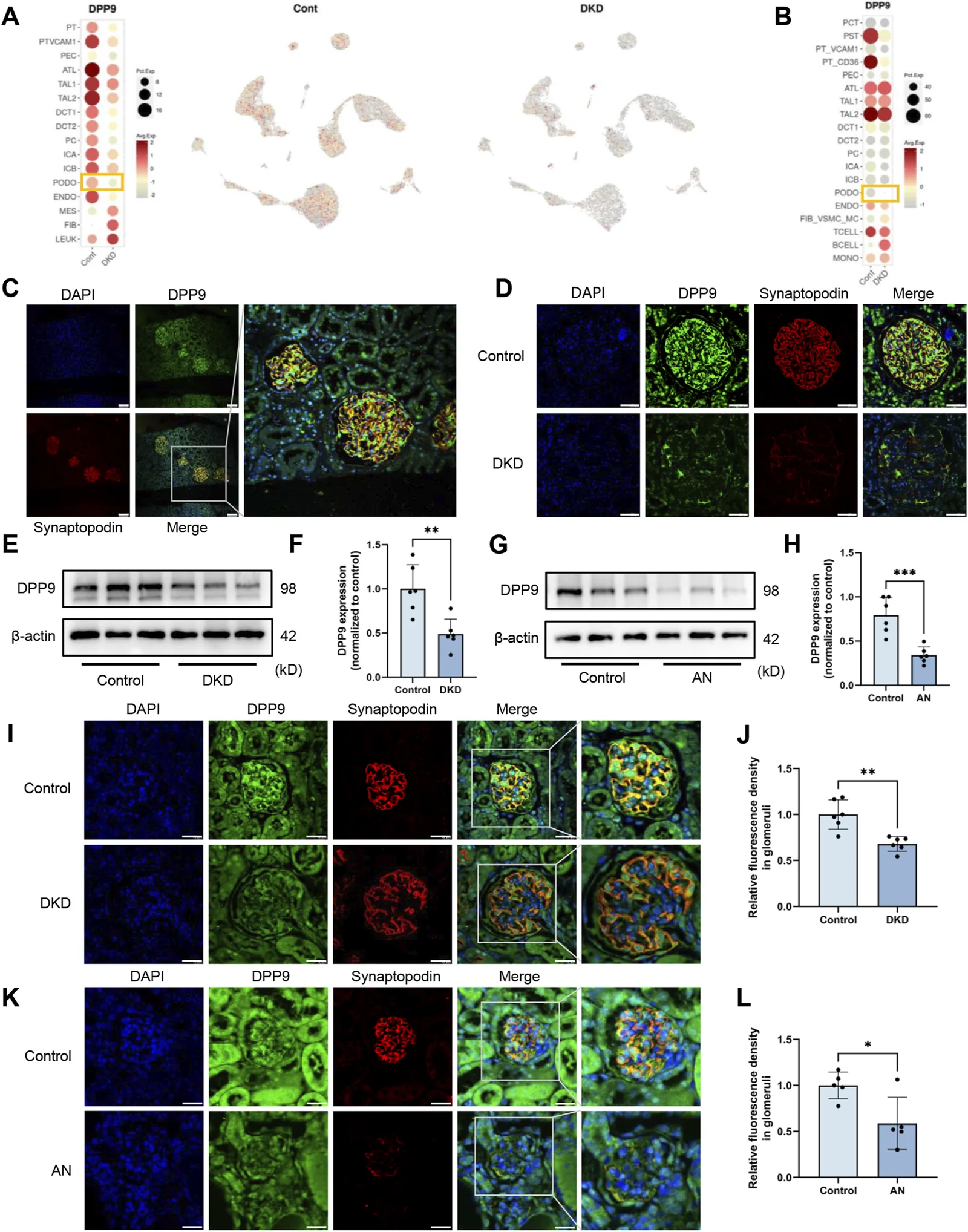
DPP9 is downregulated in injured podocytes. **(A)** DPP9 gene expression in human kidney cells via single cell analysis from the KIT database; **(B)** DPP9 gene activity and accessibility profiles in human kidney cells from the KIT database; **(C)** Representative DPP9 expression in human renal biopsy tissues (DAPI, blue; DPP9, green; Synaptopodin, red; Scale bar = 100 μm); **(D)** Representative DPP9 expression in both normal tumor-adjacent kidney tissue (control) and diabetic kidney disease (DKD) biopsy tissues (DAPI, blue; DPP9, green; Synaptopodin, red; Scale bar = 50 μm); **(E,F)** WB analysis of DPP9 protein levels in renal cortex of DKD mouse models and quantitative analysis results; **(G,H)** WB analysis of DPP9 protein levels in renal cortex of adriamycin-induced nephropathy (AN) mouse models and quantitative analysis results; **(I,J)** Representative immunofluorescence images of DPP9 in kidney of DKD mouse models and quantitative analysis results of DPP9 protein levels in glomeruli (DAPI, blue; DPP9, green; Synaptopodin, red; Scale bar = 20 μm); **(K,L)** Representative immunofluorescence images of DPP9 in kidney of AN mouse models and quantitative analysis results of DPP9 protein levels in glomeruli (DAPI, blue; DPP9, green; Synaptopodin, red; Scale bar = 20 μm). Data are presented as mean ± SD, *p < 0.05, **p < 0.01, ***p < 0.001.

### 3.2 DPP9 knockdown exacerbates podocyte injury

In *in vitro* experiments, HPC cells were used to establish podocyte injury models induced by HG or ADR. *DPP9* mRNA level was significantly decreased in HPC cells stimulated with either HG or ADR (Figure 2A). DPP9 protein levels were also decreased significantly, as shown by WB (Figures 2B–E) and cellular immunofluorescence (Figures 2F,G). These results were consistent with our *in vivo* findings for DPP9. To investigate the function of DPP9 in podocyte injury, *DPP9* expression was modulated and its impact on podocytes was evaluated. Stable DPP9 knockdown cell line was achieved using shRNA lentivirus (Supplementary Figure S2A). The shRNA #561 showed the best efficiency in downregulating DPP9 expression compared with shNC (Supplementary Figure S2A), and this cell was selected for subsequent experiments. In DPP9-knockdown HPC cells, BCL-2 protein level was reduced significantly, while BAX and Cleaved Caspase-3 were increased, suggesting that DPP9 might be involved in apoptosis (Figures 2H,I). Moreover, ADR-induced cytotoxicity was exacerbated by DPP9 knockdown (Figure 2J). Since Synaptopodin and Podocin are essential cytoskeletal proteins that are disrupted and reduced upon podocyte injury (Sun et al., 2025), they were used as markers to assess podocyte injury. The HG- or ADR-induced reductions in Synaptopodin and Podocin were amplified in DPP9-knockdown cells (Figures 2K–N), indicating that decreased DPP9 participates in podocyte cytoskeleton protein balance and aggravates podocyte injury.

**FIGURE 2:**
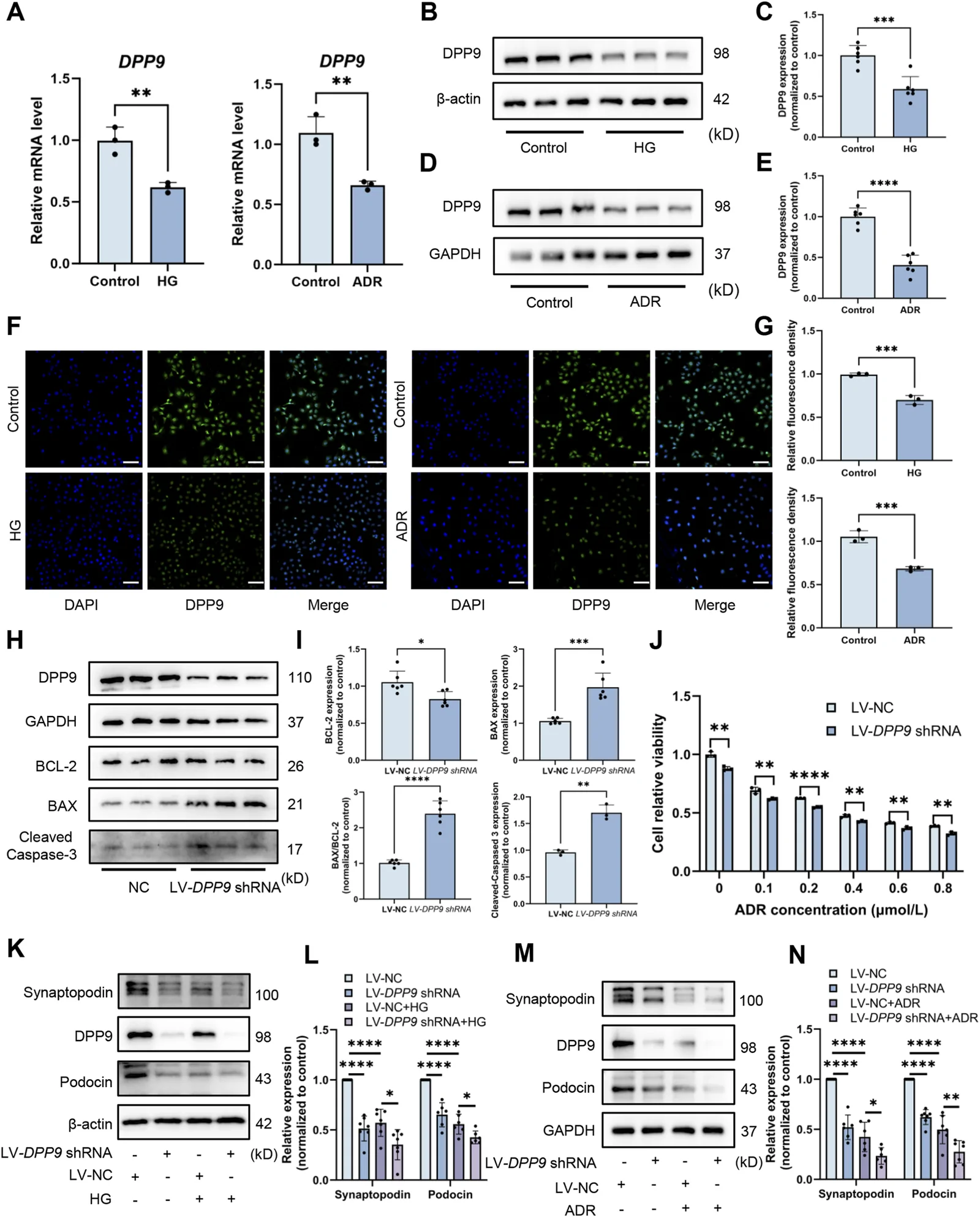
DPP9 knockdown exacerbates podocyte injury. **(A)** *DPP9* mRNA levels in high glucose (HG) or adriamycin (ADR) treated podocytes via qRT-PCR; **(B,C)** WB analysis of DPP9 protein levels in podocytes stimulated with HG and quantitative analysis results; **(D,E)** WB analysis of DPP9 protein levels in podocytes stimulated with ADR and quantitative analysis results; **(F,G)** Representative immunofluorescence images of DPP9 in podocytes stimulated with HG or ADR and quantitative analysis results of DPP9 protein levels (DAPI, blue; DPP9, green; Scale bar = 100 μm); **(H,I)** WB analysis and quantification of apoptosis-related proteins (BCL-2, BAX, Cleaved Caspase-3) levels in DPP9-knockdown HPC cells. **(J)** CCK-8 assay showing cell viability after ADR treatment in negative control (NC) and *DPP9*-knockdown podocytes; **(K,L)** WB analysis of Synaptopodin, Podocin, and DPP9 protein levels in DPP9-knockdown podocytes stimulated with HG and quantitative analysis results; **(M,N)** WB analysis of Synaptopodin, Podocin, and DPP9 protein levels in DPP9-knockdown podocytes stimulated with ADR and quantitative analysis results. Data are presented as mean ± SD. *p < 0.05, **p < 0.01, ***p < 0.001, ****p < 0.0001.

### 3.3 DPP9 modulates NRF2 activation to participate in podocyte injury

Given the known role of DPP9 in competitively binding KEAP1 to inhibit NRF2 ubiquitin-mediated degradation (Chang et al., 2023; Zhou et al., 2024), the NRF2/ARE signaling was assessed. NRF2 protein levels were reduced in DPP9-knockdown cells (Figures 3A,B and Supplementary Figure S2A), and NRF2 target genes (*HMOX1*, *GCLM*, and *AKR1C1*) were also downregulated (Figure 3C). In ADR- or HG-treated HPC cells, NRF2 protein levels were decreased, and this reduction was further exacerbated in DPP9-knockdown cells (Figures 3D–G). To evaluate the redox effect of DPP9 in podocytes, DPP9 was overexpressed in HPC cells (Figures 3H,I), and the NRF2 target genes (*HMOX1*, *GCLM*, and *AKR1C1*) were also upregulated in DPP9-overexpression cells (Figure 3J). The cell viability was improved in DPP9 overexpressed HPC cells after ADR treatment (Figure 3K). The effect of DPP9 on redox homeostasis was assessed in injured podocytes. Intracellular ROS levels were significantly increased by HG or ADR stimulation, while the HG- or ADR-triggered ROS accumulation could be attenuated by DPP9 overexpression (Figures 3L,M). The protein levels of NRF2, Synaptopodin and Podocin were evaluated in DPP9-overexpressed HPC cells following ADR or HG treatments (Figures 3N–Q). Synaptopodin and Podocin protein levels were not altered by DPP9 overexpression alone. However, the ADR- or HG-induced reductions in NRF2, Synaptopodin, and Podocin were significantly rescued in DPP9-overexpressing cells. These results indicated that overexpressed DPP9 protected podocytes from ADR- or HG-induced injury by modulating the NRF2 related antioxidation.

**FIGURE 3:**
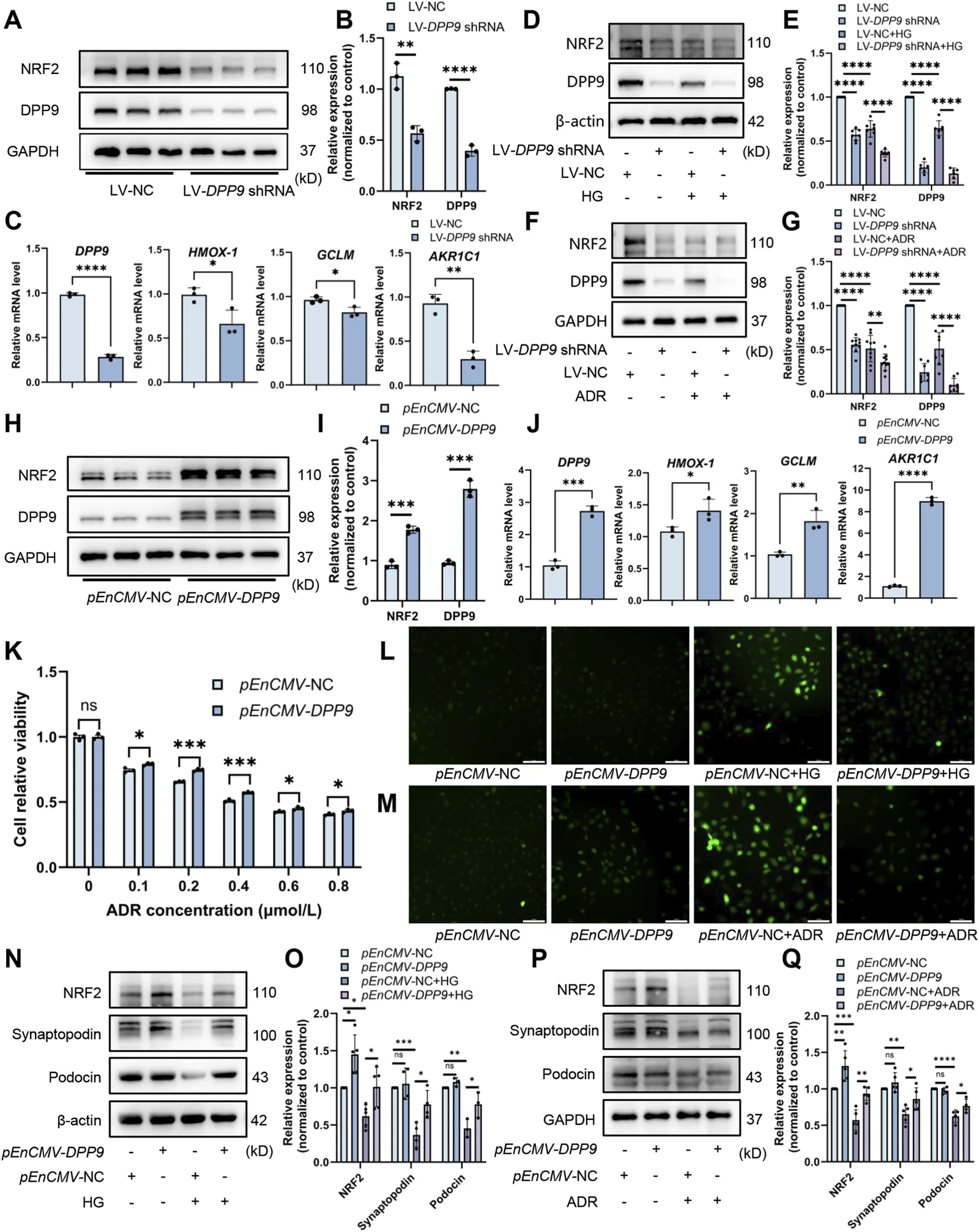
Modulation of DPP9 regulates podocyte injury via NRF2 antioxidant signaling. **(A,B)** WB analysis and quantification of DPP9 and NRF2 protein levels in HPC cells transfected with DPP9 shRNA; **(C)** qRT-PCR analysis of NRF2 target genes (*HMOX1, GCLM, and AKR1C1*) in *DPP9*-knockdown podocytes; **(D,E)** WB analysis of NRF2 and DPP9 protein levels in DPP9-knockdown podocytes stimulated with HG and quantitative analysis results; **(F,G)** WB analysis of NRF2 and DPP9 protein levels in DPP9-knockdown podocytes stimulated with ADR and quantitative analysis results; **(H,I)** WB analysis of DPP9 and NRF2 protein levels in HPC cells transfected with a DPP9 overexpression plasmid, and quantitative analysis results; **(J)** qRT-PCR analysis of NRF2 target genes (*HMOX1, GCLM, and AKR1C1*) in DPP9-overexpressing podocytes; **(K)** CCK-8 assay showing cell viability after adriamycin (ADR) treatment in negative control (NC) and DPP9-overexpressing podocytes; **(L)** Representative immunofluorescence images of ROS levels in NC and DPP9-overexpressing podocytes stimulated with HG (detected by DCFH-DA staining, green; Scale bar = 100 μm); **(M)** Representative immunofluorescence images of ROS levels in NC and DPP9-overexpressing podocytes stimulated with ADR (detected by DCFH-DA staining, green; Scale bar = 100 μm); **(N,O)** WB analysis of Synaptopodin, Podocin, and NRF2 protein levels in DPP9-overexpressing podocytes stimulated with HG and quantitative analysis results; **(P,Q)** WB analysis of Synaptopodin, Podocin, and NRF2 protein levels in DPP9-overexpressing podocytes stimulated with ADR and quantitative analysis results. Data are presented as mean ± SD. *p < 0.05, **p < 0.01, ***p < 0.001, ****p < 0.0001.

### 3.4 Finerenone modulates multiple pathways in podocytes revealed by RNA seq analysis

To assess the protective effect of finerenone on podocytes, its target MR was detected in podocytes (Supplementary Figures S3A,B). RNA-seq was used to identify differentially expressed genes (DEGs) between finerenone-treated (Finerenone group) and control (Control group) HPC cells to systematically explore the effects of finerenone on podocytes. PCA showed distinct clustering between the two groups (Figure 4A), indicating significant overall differences in the gene expression profiles. These differences were further visualized by volcano plot (Figure 4B) and hierarchical clustering heatmap (Figure 4C). Based on the screening criteria of |log_2_ FC| ≥ 1.5 and P < 0.05, a total of 716 significant DEGs were identified, including 360 upregulated genes and 356 downregulated genes. These results confirm the targeted regulatory characteristics of finerenone on podocyte gene expression. The core physiological functions of podocytes rely on key biological processes such as maintenance of the glomerular filtration barrier, dynamic balance of the cytoskeleton, regulation of ion transport, immune-inflammatory response, and metabolic homeostasis (Loreth et al., 2025). To clarify the functional localization of DEGs, GO functional enrichment analysis and KEGG pathway enrichment analysis were performed (Figures 4D,E). GO analysis revealed that DEGs were enriched in terms including glomerular capillary formation, antigen processing and presentation, metabolic regulation, cytoskeleton, and calcium ion transport complexes, covering multiple dimensions of podocyte core functions. KEGG analysis indicated that DEGs were mainly enriched in cytoskeleton-related pathways, core signaling pathways regulating podocyte function (Notch signaling pathway, Calcium signaling pathway, cAMP signaling pathway). Additionally, DEGs were enriched in pathways associated with high-risk diseases leading to podocyte injury, including systemic lupus erythematosus and diabetes. GSEA further revealed the regulatory trends of finerenone on specific functional gene sets (Figure 4F). The results demonstrated that finerenone could negatively regulate the Rho GTPase-mediated actin cytoskeleton regulatory pathway (Figure 4G) and the caspase cascade-mediated cell death pathway (Figure 4H), while inhibiting immune-inflammatory-related gene sets. Together, these findings reveal that finerenone regulates podocytes through multiple pathways, further confirming its protective effect.

**FIGURE 4:**
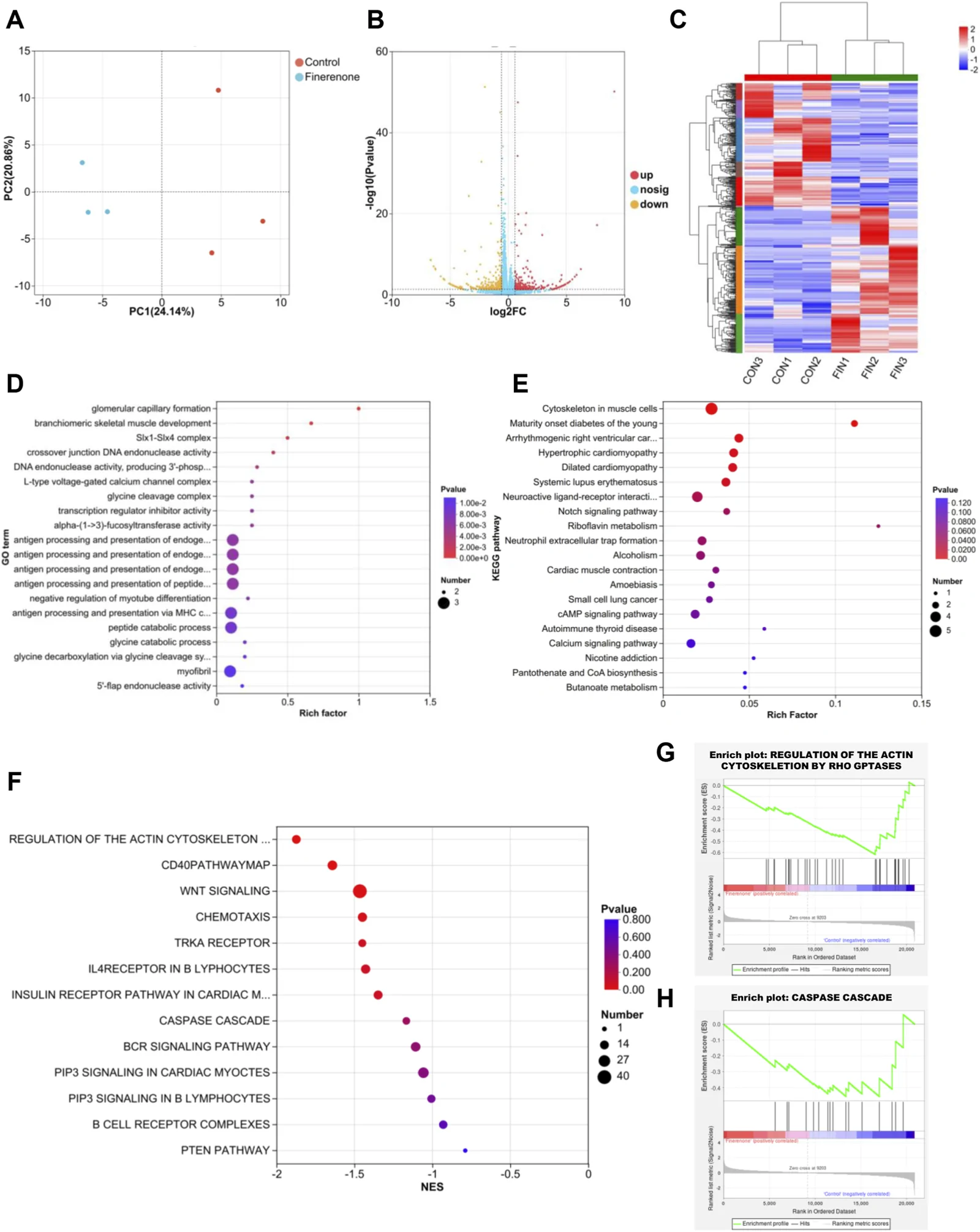
Finerenone modulates multiple pathways in podocytes revealed by RNA-seq analysis. **(A)** Principal component analysis (PCA) plot of genes between finerenone-treated and control HPC cells. **(B)** Volcano plot of differentially expressed genes (DEGs) (|log_2_FC| ≥ 1.5, *P* < 0.05). **(C)** Hierarchical clustering heatmap of significant DEGs. **(D,E)** Bubble plots of GO **(D)** and KEGG **(E)** enrichment analyses for DEGs. **(F)** GSEA bubble plot showing enrichment of functional gene sets in finerenone-treated cells. **(G,H)** GSEA enrichment plots of *REGULATION OF THE ACTIN CYTOSKELETON BY RHO GTPases* **(G)** and *CASPASE CASCADE* **(H)** gene sets in finerenone-treated HPC cells.

### 3.5 Finerenone upregulates DPP9 and NRF2 to protect podocytes against injury

DPP9 has been reported to play critical roles in small GTPase-mediated cytoskeletal rearrangement (Zhao et al., 2025), caspase-1-mediated pyroptosis, cell adhesion and migration, antigen presentation, and energy metabolism (Cui et al., 2022)—all of which are consistent with the regulatory pathways of finerenone identified by RNA-seq. Furthermore, given prior evidence that aldosterone promotes oxidative stress and inhibits the NRF2 pathway, whereas finerenone ameliorates oxidative stress (Koçak et al., 2025; Gaikwad et al., 2022), we hypothesized that finerenone could regulate DPP9 to enhance NRF2-mediated antioxidant responses and protect podocytes from injury. HPC cells were treated with different doses of finerenone and cell viability was assessed. We found 4 μM of finerenone did not affect cell viability, whereas 8 μM of finerenone significantly reduced cell viability (Supplementary Figure S3C). Therefore, the non-cytotoxic concentration of 4 μM was selected for subsequent experiments. Furthermore, the protein levels of DPP9 (Figures 5A,B; Supplementary Figures S3D,E) and NRF2 were significantly increased (Figures 5A,B) in finerenone-treated HPC cells. The mRNA expression of NRF2 target genes (*HMOX1*, *GCLM*, and *AKR1C1*) was also significantly enhanced by finerenone treatment (Figure 5C). Finerenone protected podocytes from ADR-induced loss of cell viability (Figure 5D). Both HG and ADR could also cause oxidative stress in podocytes. Here we showed that HG- or ADR-induced ROS accumulation was suppressed by finerenone (Figures 5E–H). In addition, finerenone restored the protein levels of DPP9, NRF2, Synaptopodin and Podocin in HG- or ADR-induced HPC cells (Figures 5I–L). These results suggest that finerenone may protect podocytes from oxidative injury by upregulating DPP9 and activating NRF2.

**FIGURE 5:**
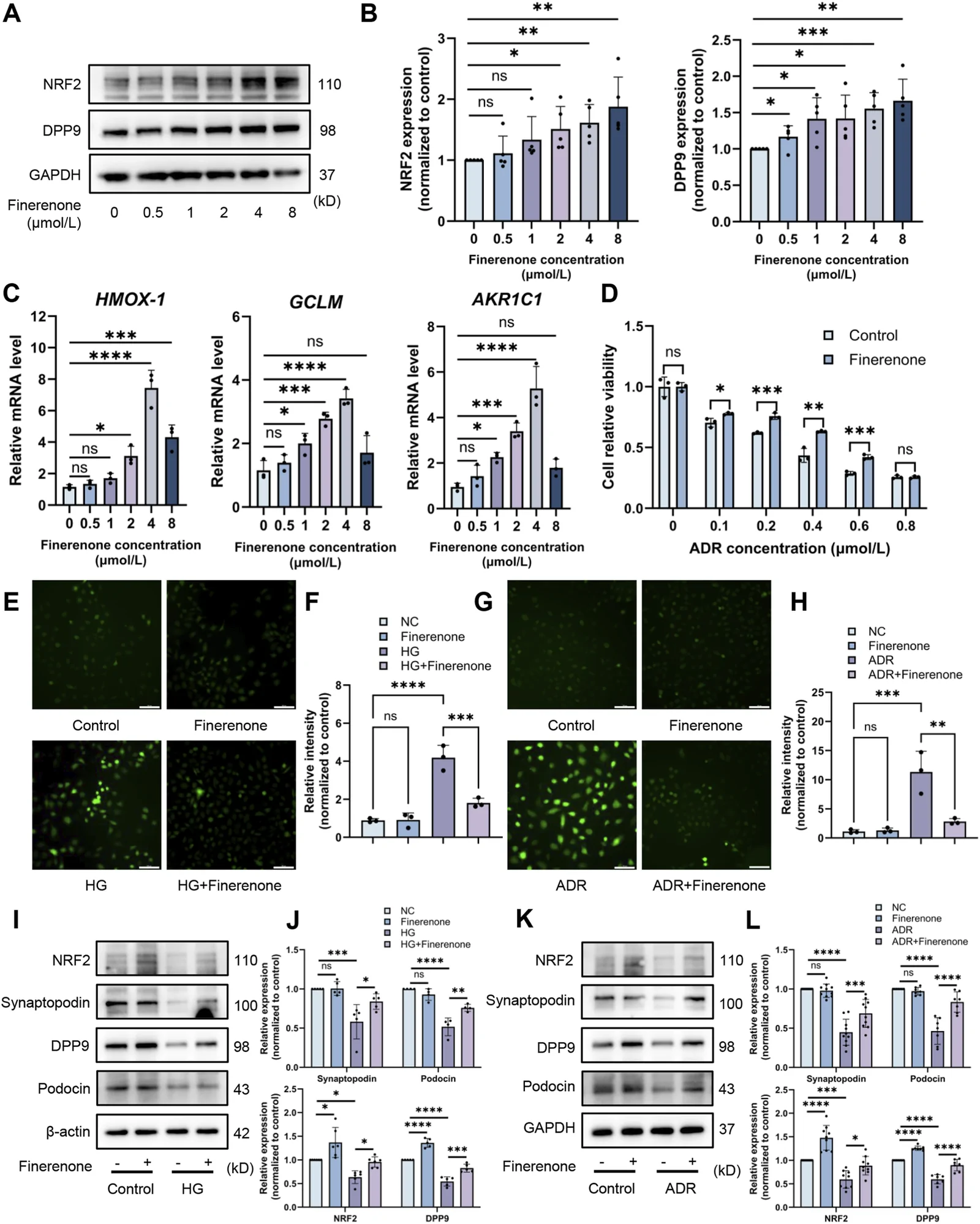
Finerenone upregulates DPP9, activates NRF2, attenuates oxidative stress, and exerts podocyte protection. **(A,B)** WB analysis and quantification of DPP9 and NRF2 protein levels in HPC cells treated with different concentrations of finerenone (0.5, 1, 2, 4, 8 μM); **(C)** qRT-PCR analysis of NRF2 target genes (*HMOX1, GCLM, and AKR1C1*) in podocytes treated with 4 μM finerenone; **(D)** CCK-8 assay showing the effect of finerenone treatment on ADR-induced podocyte viability changes; **(E,F)** Representative immunofluorescence images and quantitative analysis of ROS levels in finerenone-treated podocytes stimulated with HG (detected by DCFH-DA staining, green; Scale bar = 100 μm); **(G,H)** Representative immunofluorescence images and quantitative analysis of ROS levels in finerenone-treated podocytes stimulated with ADR (detected by DCFH-DA staining, green; Scale bar = 100 μm); **(I,J)** WB analysis and quantification of the effect of finerenone treatment on protein levels of Synaptopodin, Podocin, NRF2, and DPP9 in HG-stimulated podocytes; **(K,L)** WB analysis and quantification of the effect of finerenone treatment on protein levels of Synaptopodin, Podocin, NRF2, and DPP9 in ADR-stimulated podocytes. Data are presented as mean ± SD. *p < 0.05, **p < 0.01, ***p < 0.001, ****p < 0.0001.

### 3.6 Finerenone exerts podocyte protection via DPP9/NRF2 axis

To evaluate the causal role of DPP9 in finerenone-mediated protection, DPP9-knockdown HPC cells were used. NRF2 protein levels failed to be upregulated by finerenone treatment in DPP9-knockdown HPC cells (Figures 6A,B). Consistently, the enhanced effects of finerenone on the NRF2 target genes (*HMOX1*, *GCLM*, and *AKR1C1*) were also attenuated or abolished in DPP9 knockdown HPC cells (Figure 6C). The protective effect of finerenone on cell viability was also abolished in DPP9-knockdown HPC cells under ADR treatment (Figure 6D). Meanwhile, the ability of finerenone to mitigate HG- or ADR-induced reductions in Synaptopodin and Podocin, as well as the suppression of NRF2, was attenuated in DPP9-knockdown cells (Figures 6E–H). KEAP1-knockdown HPC cells were established to assess the role of KEAP1 in finerenone-induced NRF2 activation. The result showed that finerenone failed to induce NRF2 activation in KEAP1-knockdown HPC cells (Figures 6I,J). Additionally, finerenone significantly upregulated DPP9 and NRF2 protein levels, enhanced DPP9-KEAP1 binding, and decreased NRF2-KEAP1 binding (Figures 6K,L). Furthermore, we found that finerenone reduced NRF2 ubiquitination in a DPP9-dependent manner (Figure 6M). These results suggest that finerenone protects podocytes by upregulating DPP9, which enhances NRF2 protein stability and its antioxidant effect.

**FIGURE 6:**
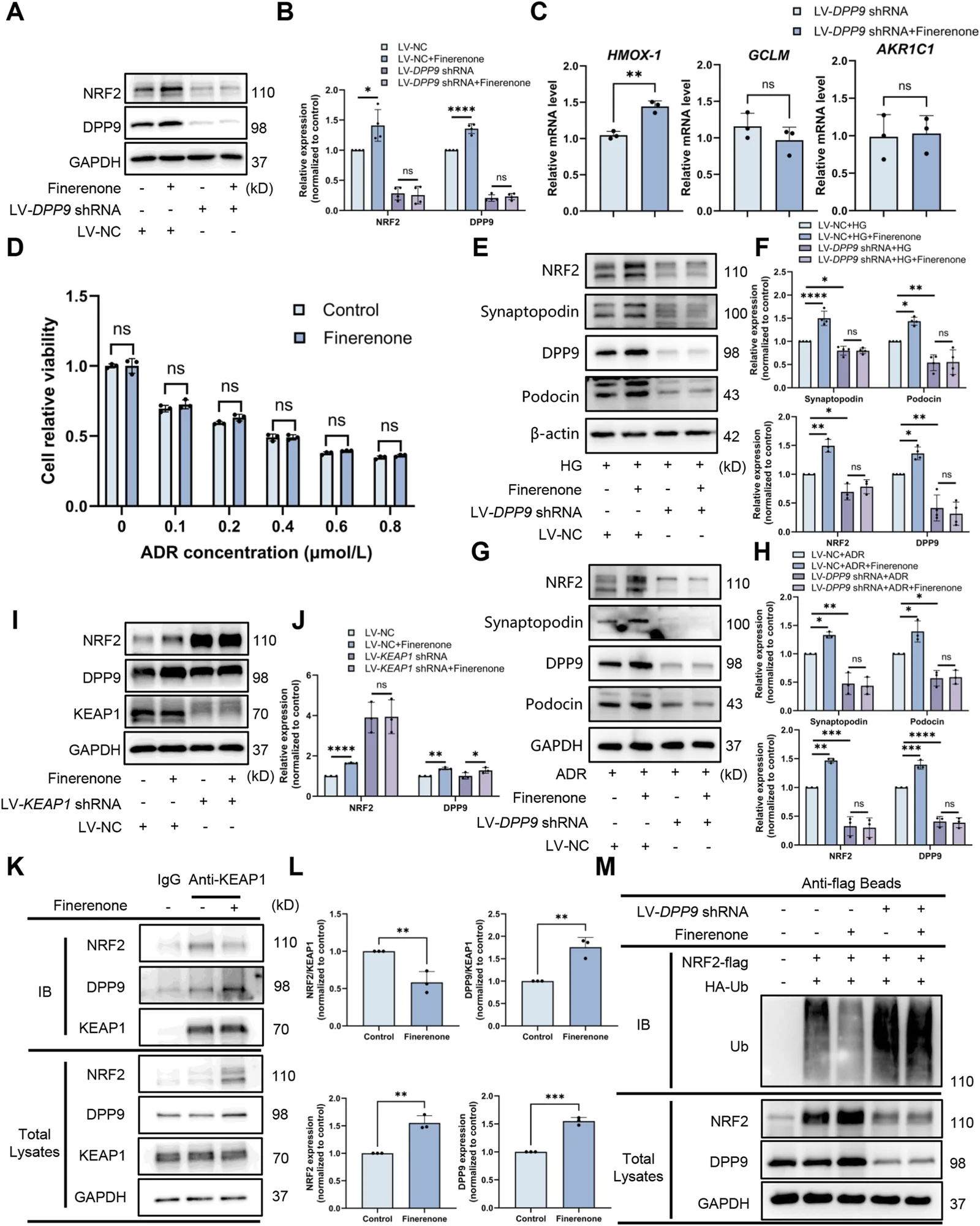
Finerenone activates NRF2 by upregulating DPP9 to attenuate KEAP1-NRF2 interaction. **(A,B)** WB analysis and quantification of protein levels of NRF2 and DPP9 in finerenone-treated podocytes with or without DPP9 knockdown; **(C)** qRT-PCR analysis of NRF2 target genes (*HMOX1, GCLM, and AKR1C1*) in DPP9-knockdown podocytes treated with finerenone; **(D)** CCK-8 assay showing the effect of finerenone on ADR-induced viability changes in DPP9-knockdown podocytes; **(E,F)** WB analysis and quantification of the effect of finerenone treatment on protein levels of Synaptopodin, Podocin, NRF2, and DPP9 in HG-stimulated DPP9-knockdown podocytes; **(G,H)** WB analysis and quantification of the effect of finerenone treatment on protein levels of Synaptopodin, Podocin, NRF2, and DPP9 in ADR-stimulated DPP9-knockdown podocytes; **(I,J)** WB analysis of protein levels of NRF2, DPP9, and KEAP1 in finerenone-treated podocytes with or without KEAP1 knockdown; **(K,L)** Immunoprecipitation assay evaluating the effect of finerenone on the binding between KEAP1 and DPP9 or NRF2; **(M)** Immunoprecipitation assay evaluating the ubiquitination-induced degradation of NRF2 in finerenone-treated podocytes with or without DPP9 knockdown. Data are presented as mean ± SD. *p < 0.05, **p < 0.01, ***p < 0.001, ****p < 0.0001.

### 3.7 Finerenone upregulates renal DPP9/NRF2 and alleviates kidney injury

To evaluate the finerenone protective effect on podocytes *in vivo*, AN mouse model was used. The experimental protocol is shown in Figure 7A. Consistent with the *in vitro* findings, increased DPP9 and NRF2 protein levels in the renal cortex of mice were induced by finerenone (Figures 7B,C). Additionally, DPP9 expression was upregulated in the glomeruli of AN mice after finerenone treatment (Figures 7D,E). In the AN group, plasma creatinine levels and urinary albumin-to-creatinine ratio (ACR) were significantly increased compared with the control group, whereas no differences were observed between the control and finerenone groups. More importantly, both plasma creatinine and uACR were significantly ameliorated in AN + finerenone group (Figure 7F). Consistent with these biochemical alterations, severe renal damage in the AN group—characterized by tubular dilation, extensive atrophy, proteinaceous cast formation, interstitial fibrosis, and glomerulosclerosis—was revealed by histopathological analyses (H&E staining, Masson’s trichrome staining, and PAS staining) (Figures 7G,H). Notably, these pathological alterations were significantly attenuated by finerenone treatment (Figures 7G,H). In addition, finerenone mitigated the disruption of Synaptopodin and the upregulation of Desmin expression in the glomeruli of AN mice (Figures 7I,J). Finerenone also reduced podocyte loss in AN mice (Figures 7K,L). These results confirm that finerenone upregulates DPP9 and NRF2, protects podocytes, and alleviates kidney injury *in vivo*.

**FIGURE 7:**
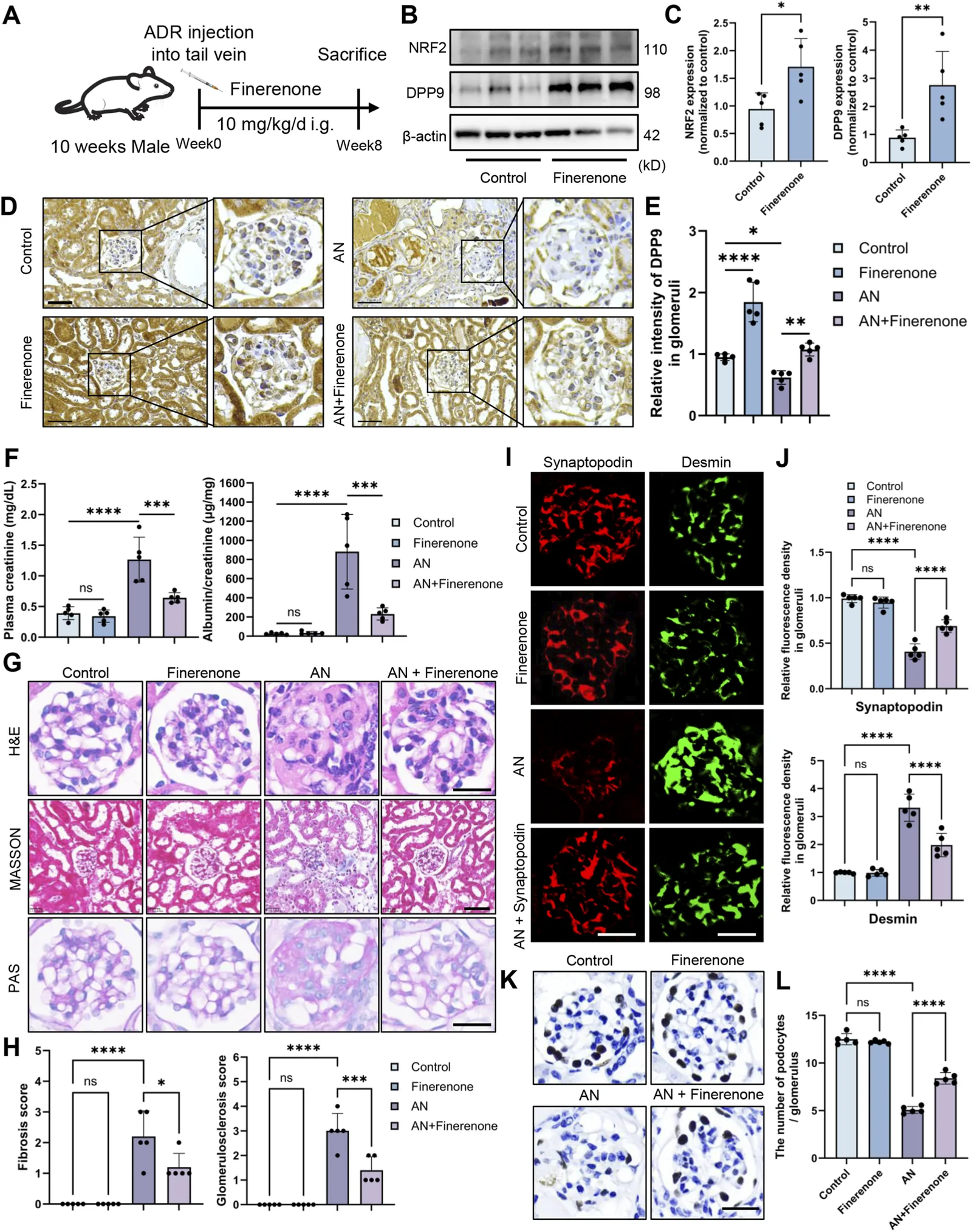
Finerenone upregulates renal DPP9/NRF2 and ameliorates adriamycin (ADR)-induced podocyte injury in mice. **(A)** Schematic diagram of ADR-induced podocyte injury model establishment and finerenone intervention (gavage, 10 mg/kg/day) in mice (Groups: Control group, Finerenone group, ADR group, ADR + Finerenone group); **(B,C)** WB analysis of DPP9 and NRF2 protein levels in the renal cortex of mice in different groups and quantitative analysis results; **(D,E)** Representative IHC images of DPP9 in mouse kidneys and quantitative analysis results of DPP9 protein levels in glomeruli from mice (Scale bar = 50 μm); **(F)** Plasma creatinine levels and urinary albumin-to-creatinine ratio (ACR) in mice of different groups; **(G)** Representative images of H&E, Masson’s trichrome, and PAS staining in kidney sections from different groups. (Scale bar = 25 μm); **(H)** Quantitative analysis results of interstitial fibrosis and glomerulosclerosis score in different groups; **(I,J)** Representative immunofluorescence images and quantitative analysis results of Synaptopodin and Desmin in glomeruli from mice (Synaptopodin, red; Desmin, green; Scale bar = 20 μm); **(K,L)** Representative IHC images and quantifications of Wilms’ Tumor 1 (WT-1) per glomerulus in glomeruli from mice (Scale bar = 20 μm). Data are presented as mean ± SD. *p < 0.05, **p < 0.01, ***p < 0.001, ****p < 0.0001.

## 4 Discussion

In this study, we confirmed the protective effect of DPP9 in podocyte injury. In both KIT database and clinical kidney biopsies of DKD patients, we demonstrated that DPP9 was significantly decreased in injured podocytes. This finding was validated in mouse and cell models of podocyte injury. Finerenone could upregulate DPP9 protein levels in podocytes and protect against podocyte injury in AN model. We also showed that upregulated DPP9 in podocytes could induce NRF2 activation via competitively binding to KEAP1 and reducing NRF2 ubiquitination. Collectively, these results suggest that finerenone-upregulated DPP9 protects injured podocytes through its antioxidative effects, by suppressing ubiquitin-mediated degradation of NRF2.

Podocyte injury is a key driver of CKD progression, leading to proteinuria and reduced glomerular filtration rate (Shankland, 2006; Sever and Schiffer, 2018). Therefore, identifying the key molecules that maintain podocyte structure and function is essential for delaying kidney disease progression. Here, we found that reduced DPP9 levels in podocytes from DKD patients, animal models, and cellular injury models would be an important factor contributing to podocyte injury. DPP9 knockdown exacerbated the reduction of Synaptopodin and Podocin in injured podocytes. Conversely, overexpressed DPP9 exerted a podocyte-protective effect, as evidenced by its ability to restore the reductions in Synaptopodin and Podocin-the two essential cytoskeletal proteins that maintain podocyte structural and functional integrity (Sun et al., 2025). DPP9 belongs to the serine protease family, which recognizes and cleaves N-terminal dipeptides from substrates with proline at the penultimate position (Cui et al., 2022). However, the non-enzymatic functions of DPP9 as an adaptor protein have garnered increasing attention in recent years, particularly its ability to mediate protein–protein interactions—such as those with NLRP1, CARD8, and KEAP1 (Chang et al., 2023; Zhou et al., 2024; Huang et al., 2021; Sharif et al., 2021; Hollingsworth et al., 2021)—implicating DPP9 in diverse cellular processes including pyroptosis and redox regulation.

Our study also demonstrated that DPP9 acts as a regulator of redox homeostasis in podocytes. DPP9 overexpression significantly attenuated HG- and ADR-induced ROS accumulation in podocytes, with the underlying mechanism involving the upregulation and activation of NRF2. As a transcription factor, NRF2 is a major regulator of intracellular oxidative stress; its activation controls the transcription of numerous antioxidant enzyme genes to counteract oxidative damage (Zhang et al., 2025; Schmidlin et al., 2019). For instance, NRF2 is crucial in DKD—podocyte-specific NRF2 knockout exacerbates renal injury in DKD models (Zhang et al., 2018). Under basal conditions, KEAP1 binds to NRF2 in a 2:1 ratio, one KEAP1 molecule binds to the ETGE motif of NRF2, while the other binds to the DLG motif, mediating NRF2 ubiquitination and degradation to regulate NRF2 levels (Tong et al., 2007). Disruption of this complex leads to increased NRF2 levels, which then translocate to the nucleus, bind to AREs, and initiate transcription of ARE-regulated genes (Nguyen et al., 2003). The level of NRF2 is regulated via multiple distinct molecular mechanisms. For instance, TEAD1 modulates NRF2 expression and exerts a crucial protective role against oxidative stress during cisplatin-induced acute kidney injury (Tran et al., 2025; Jagannathan et al., 2025). JMJD3 upregulates NRF2 by relieving epigenetic repression of NRF2 (Huang et al., 2020; An et al., 2023). Additionally, p62 can bind to KEAP1, thereby preventing KEAP1-mediated degradation of SOX4 and NRF2, which allows SOX4 and NRF2 to form a complex in the nucleus (Tsai et al., 2025; Du et al., 2026). Separately, PTEN promotes NRF2 degradation through the PI3K/AKT/GSK-3β axis in a KEAP1-independent manner (Rojo et al., 2014; An et al., 2022). Collectively, these findings highlight the sophisticated multilayered regulation governing NRF2 activity. This study found that DPP9 binds to KEAP1 and that DPP9-knockdown enhances NRF2 ubiquitination in HPC cells. Similar findings have been reported in clear cell renal cell carcinoma and hepatocellular carcinoma cells, in which DPP9 binds to the KELCH domain of KEAP1 via its conserved ESGE motif, competitively inhibiting the interaction between KEAP1 and NRF2 in an enzyme-independent manner (Chang et al., 2023; Zhou et al., 2024; Tsamouri et al., 2025). Consequently, decreased DPP9 expression in injured podocytes promotes the pathogenesis of kidney disease by suppressing NRF2 activation and disrupting redox homeostasis.

Growing efforts have been devoted to identifying podocyte-protective agents, including synthetic compounds and natural products. For example, natural derivatives such as Wogonoside have been reported to attenuate podocyte injury in diabetic nephropathy by targeting the NF-κB p65–MMP28 axis (Li X. et al., 2025). Finerenone, a novel non-steroidal MRA, confers renal protection by antagonizing MR. We confirmed that MR is expressed in podocytes. The FIDELIO-DKD trial demonstrated that finerenone significantly reduces the risk of CKD progression and cardiovascular events in patients with CKD and type 2 diabetes (Bakris et al., 2020). The FIDELITY pooled analysis, combining data from FIDELIO-DKD and FIGARO-DKD, further demonstrated that finerenone reduces the risk of kidney failure (Agarwal et al., 2022). Results from the FINEARTS-HF trial indicated that finerenone effectively reduces proteinuria in patients (Mc Causland et al., 2025). Moreover, finerenone has also shown efficacy in non-diabetic chronic kidney disease (Ma et al., 2026). These clinical studies suggest that finerenone exerts a profound effect on reducing proteinuria. Here, we demonstrated that finerenone alleviates podocyte injury and preserves renal function by upregulating DPP9 and activating NRF2 signaling.

Our study found that finerenone could significantly upregulate DPP9 and NRF2 in podocytes. Finerenone also activated NRF2 and reduced ROS accumulation in podocytes; importantly, its protective effects were diminished in DPP9-knockdown podocytes, indicating that DPP9 is involved in finerenone-mediated NRF2 regulation and podocyte protection. Mechanistically, as shown in Figure 8, finerenone upregulates DPP9, enhances DPP9-KEAP1 binding, reduces NRF2-KEAP1 interaction, which decreases NRF2 ubiquitination, and ultimately counteracts oxidative stress. These findings are consistent with previous findings that MR activation induces oxidative stress and inhibits the NRF2 pathway under pathological conditions (Nakamura et al., 2022; Gaikwad et al., 2022; Escobar Vasco et al., 2024), and that finerenone can ameliorate oxidative stress (Koçak et al., 2025; Gaikwad et al., 2022). Nevertheless, the mechanism by which finerenone upregulates DPP9 remains to be elucidated.

**FIGURE 8:**
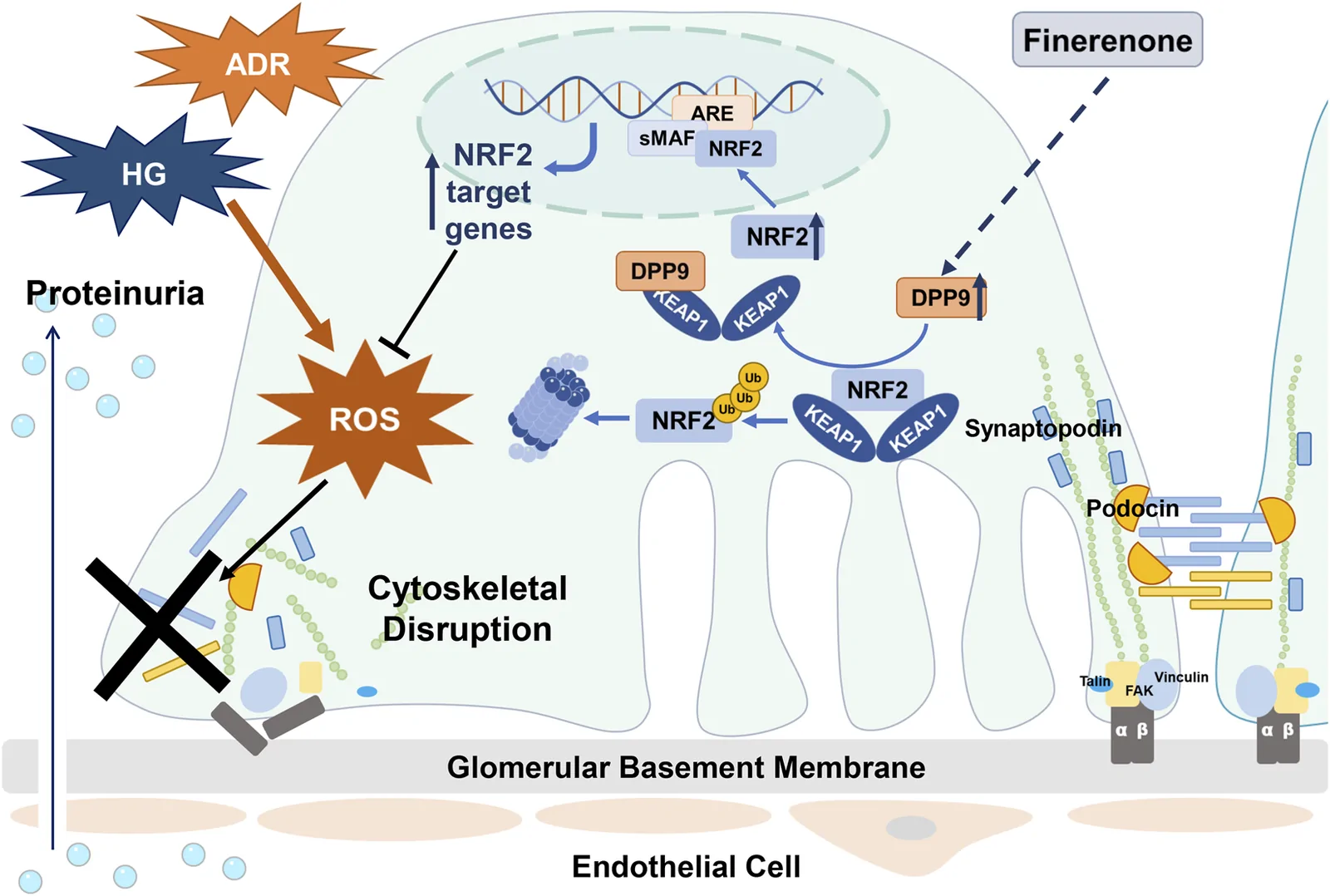
Schematic diagram of finerenone’s protective mechanism against oxidative podocyte injury. Finerenone exerts its protective effect on podocytes by upregulating DPP9, which competitively binds to KEAP1. This inhibits NRF2 ubiquitination and degradation, leading to increased NRF2 protein levels and activation of its target genes, enhancing cellular antioxidant capacity and reducing intracellular reactive oxygen species (ROS) levels. Ultimately, this process attenuates oxidative stress-induced podocyte injury.

Oxidative stress also promotes inflammatory responses by triggering DNA damage to activate the cGAS–STING pathway and inflammasome assembly (Jiao et al., 2025; Li H. et al., 2025). Previous studies have demonstrated that DPP9 participates in DNA damage repair and inflammasome regulation (Nguyen et al., 2025). NRF2 can also exert anti-inflammatory effects and regulate immune regulation; for example, STAT6 can directly activate NRF2 transcription, thereby promoting antioxidant and anti-inflammatory responses (Tu et al., 2023; Jiao et al., 2021a; Jiao et al., 2021b). Accordingly, the DPP9-NRF2 axis may exert dual renoprotective functions in kidney injury, combating both oxidative stress and inflammatory responses. Beyond oxidative stress, DPP9 and finerenone may also influence podocyte function through other key pathways. The precise regulation of the actin cytoskeleton is crucial for sustaining podocyte function (Haydak and Azeloglu, 2024). We found DPP9 knockdown could promote apoptosis and disrupt the cytoskeleton proteins Synaptopodin and Podocin in HPC cells. Finerenone could reverse cytoskeleton disruption of podocytes both *in vivo* and *in vitro*. RNA-seq analysis revealed that finerenone regulates podocyte gene expression through a multi-targeted and multi-pathway synergistic manner, primarily involving key processes such as maintaining cytoskeletal dynamic balance, inhibiting excessive cell death, regulating immune-inflammatory responses, and preserving metabolic homeostasis. Additionally, DPP9 has also been reported to directly regulate the actin polymerization pathway and to interact directly with small GTPases (such as Rac1) in the hippocampus, contributing to cytoskeletal regulation (Zhao et al., 2025). Therefore, finerenone may also regulate DPP9 to participate in podocyte protection by maintaining cytoskeletal dynamics and through other pathways.

In conclusion, this study is the first to demonstrate a critical role for DPP9 in podocyte biology. Reduced DPP9 expression impairs podocyte integrity by suppressing NRF2 activation and compromising cellular antioxidant capacity. Notably, finerenone upregulates DPP9, thereby enhancing NRF2 signaling and downstream antioxidant defenses, which represents a novel mechanistic basis for its podocyte-protective effects.

## Data Availability

The RNA-seq data generated in this study have been deposited in the Gene Expression Omnibus (GEO) under accession number GSE346384 (https://www.ncbi.nlm.nih.gov/geo/query/acc.cgi?acc=GSE346384).
