# Supplementary material for: Targeting DPP9 attenuates podocyte injury by regulating NRF2 antioxidant signaling
Source: Front Pharmacol. 2026 Sep 17;17:1930044. doi: 10.3389/fphar.2026.1930044 (PMC13627425; doi:10.3389/fphar.2026.1930044)
Supplement: Supplementary file 1 [file DataSheet1.PDF]

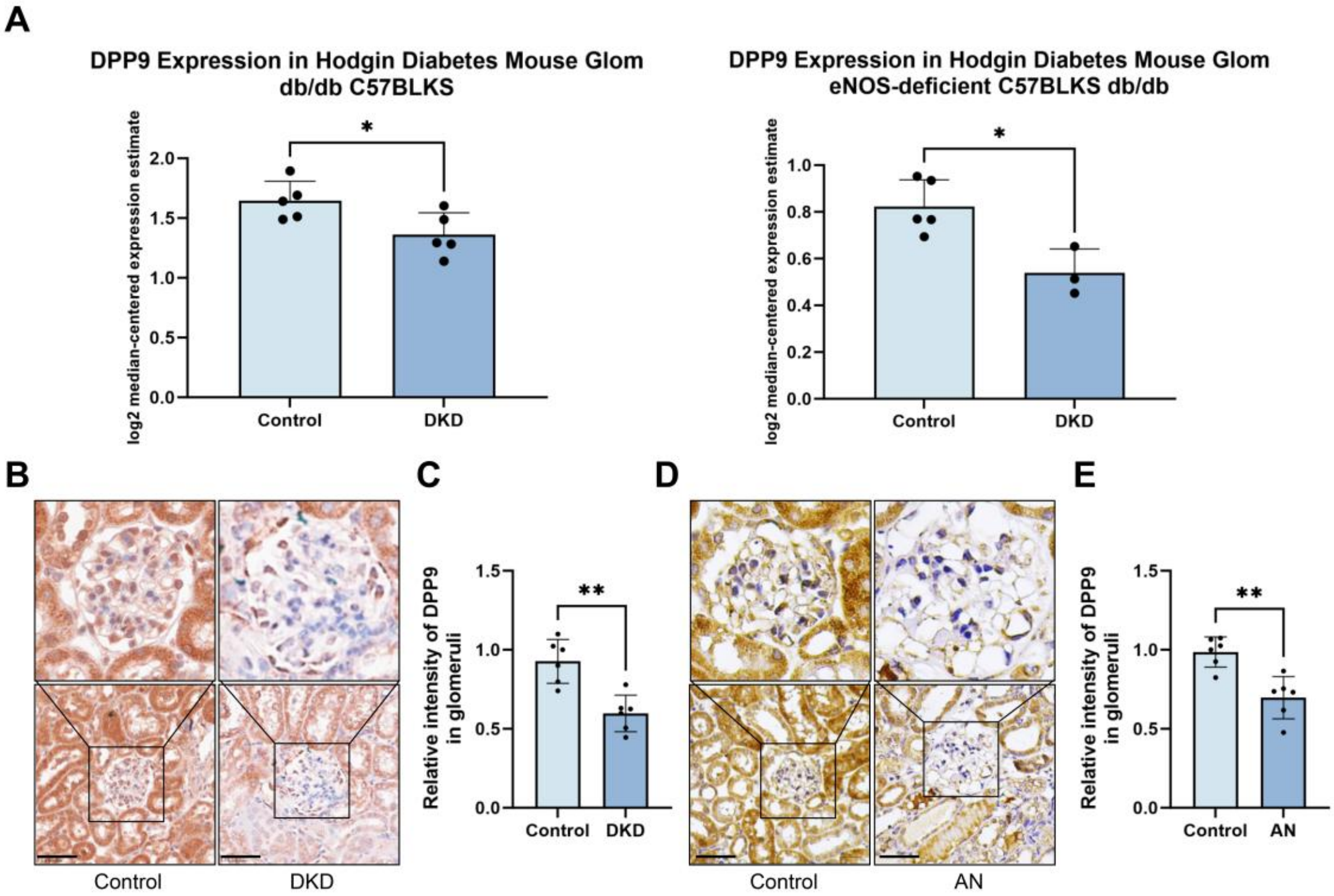

Fig. S1 Decreased DPP9 expression in glomeruli of diabetic mice and in DKD and adriamycin-induced nephropathy (AN) mouse models

(A) Relative expression level of *Dpp9* in glomeruli of diabetic mice from the NephroSeq transcriptome database (V5); (B, C) Representative IHC images of DPP9 in kidneys of DKD mouse models and quantitative analysis results of DPP9 protein levels in glomeruli (Scale bar = 50 μm); (D, E) Representative IHC images of DPP9 in kidneys of AN mouse models and quantitative analysis results of DPP9 protein levels in glomeruli (Scale bar = 50 μm). Data are presented as mean ± SD. *$p < 0.05$, **$p < 0.01$.

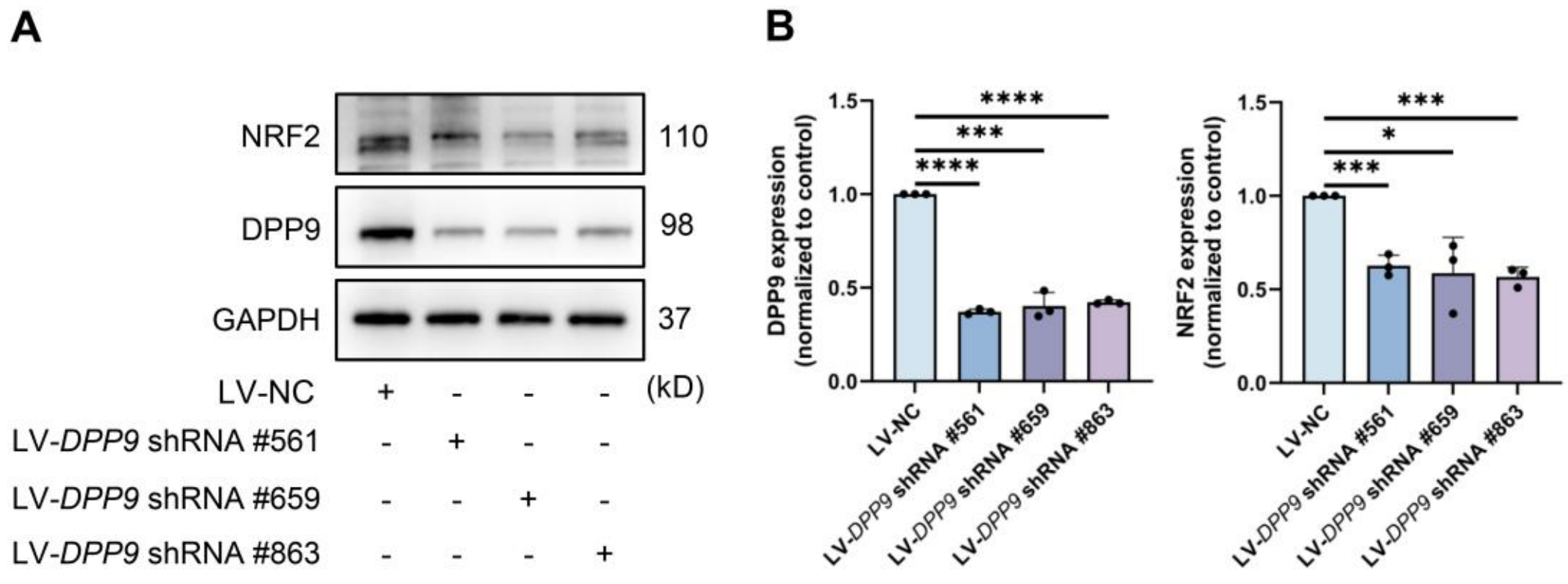

Fig. S2 Validation of DPP9 knockdown and its effects on NRF2 levels

(A, B) WB analysis and quantification of DPP9 and NRF2 protein levels in HPC cells transfected with DPP9 shRNAs (#561, #659, #863). Data are presented as mean ± SD. *$p < 0.05$, ***$p < 0.001$, ****$p < 0.0001$.

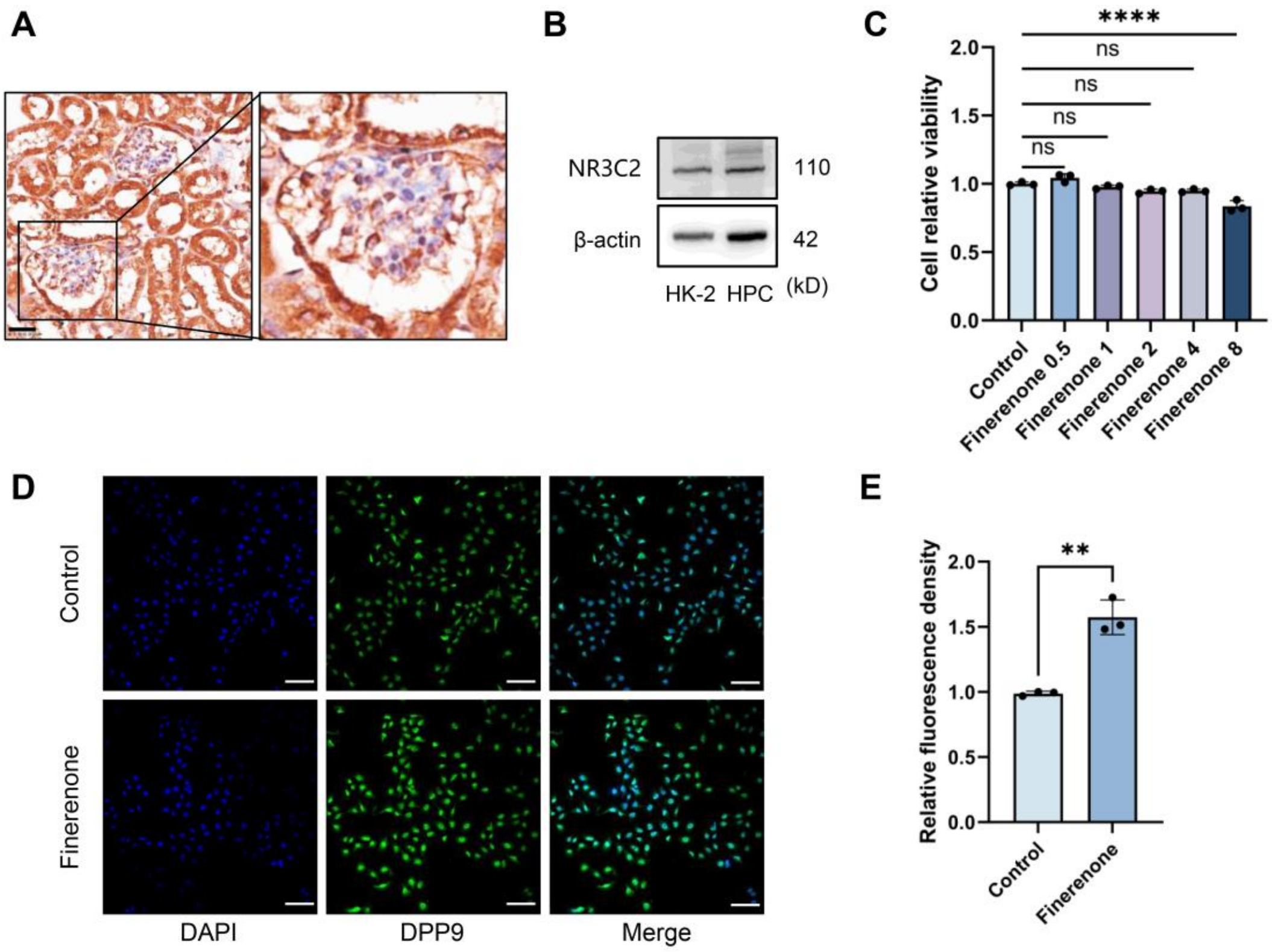

Fig. S3 Mineralocorticoid receptor (MR) expression, finerenone effects on cell viability and finerenone-mediated DPP9 regulation in podocytes

(A) Representative IHC images of mineralocorticoid receptor (MR, gene name: NR3C2) in mouse kidneys (Scale bar = 25 μm); (B) WB analysis of NR3C2 expression in human renal tubular epithelial cells (HK-2) and human podocyte cells (HPC); (C) CCK-8 assay showing cell viability changes of HPC cells treated with different concentrations of finerenone (0.5, 1, 2, 4, 8 μM); (D, E) Representative immunofluorescence images and quantitative analysis results of DPP9 in podocytes treated with finerenone (4 μM) (Scale bar = 100 μm). Data are presented as mean ± SD. $^{**}p < 0.01$, $^{****}p < 0.0001$.
