# Supplementary material for: Targeting DPP9 attenuates podocyte injury by regulating NRF2 antioxidant signaling
Source: Front Pharmacol. 2026 Sep 17;17:1930044. doi: 10.3389/fphar.2026.1930044 (PMC13627425; doi:10.3389/fphar.2026.1930044)

Fig. 1E

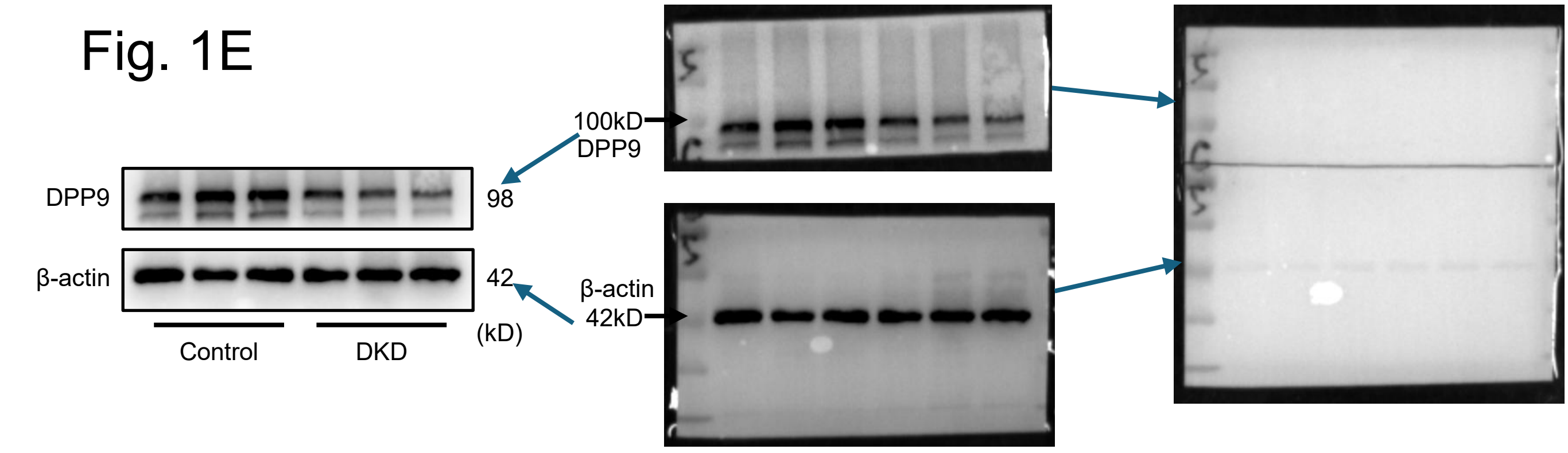

Fig. 1G

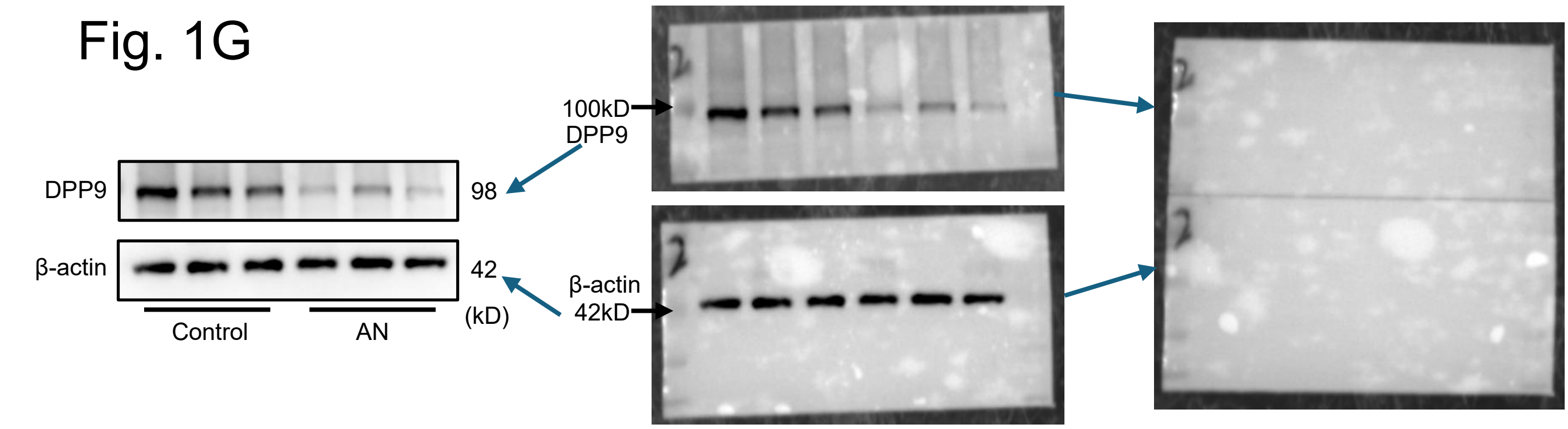

Fig. 2B

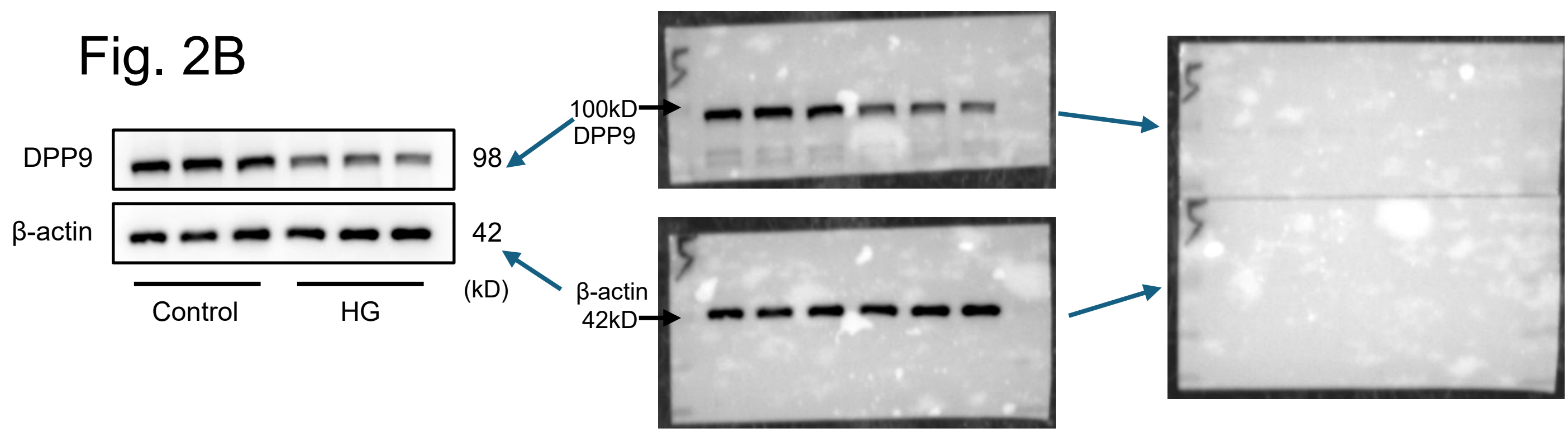

Fig. 2D

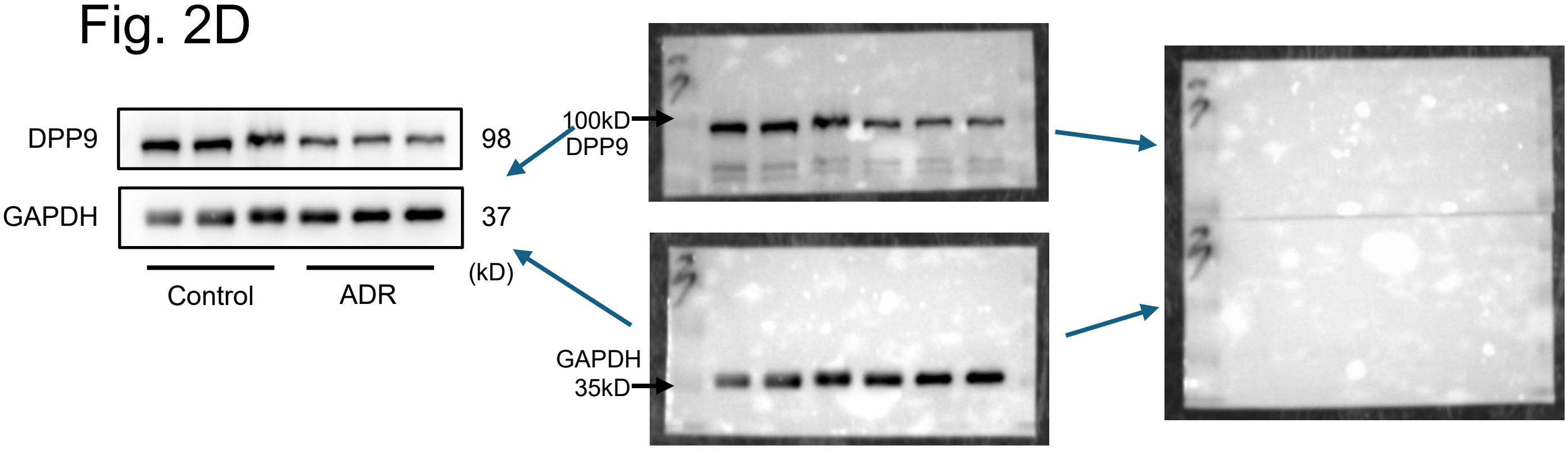

Fig. 2H

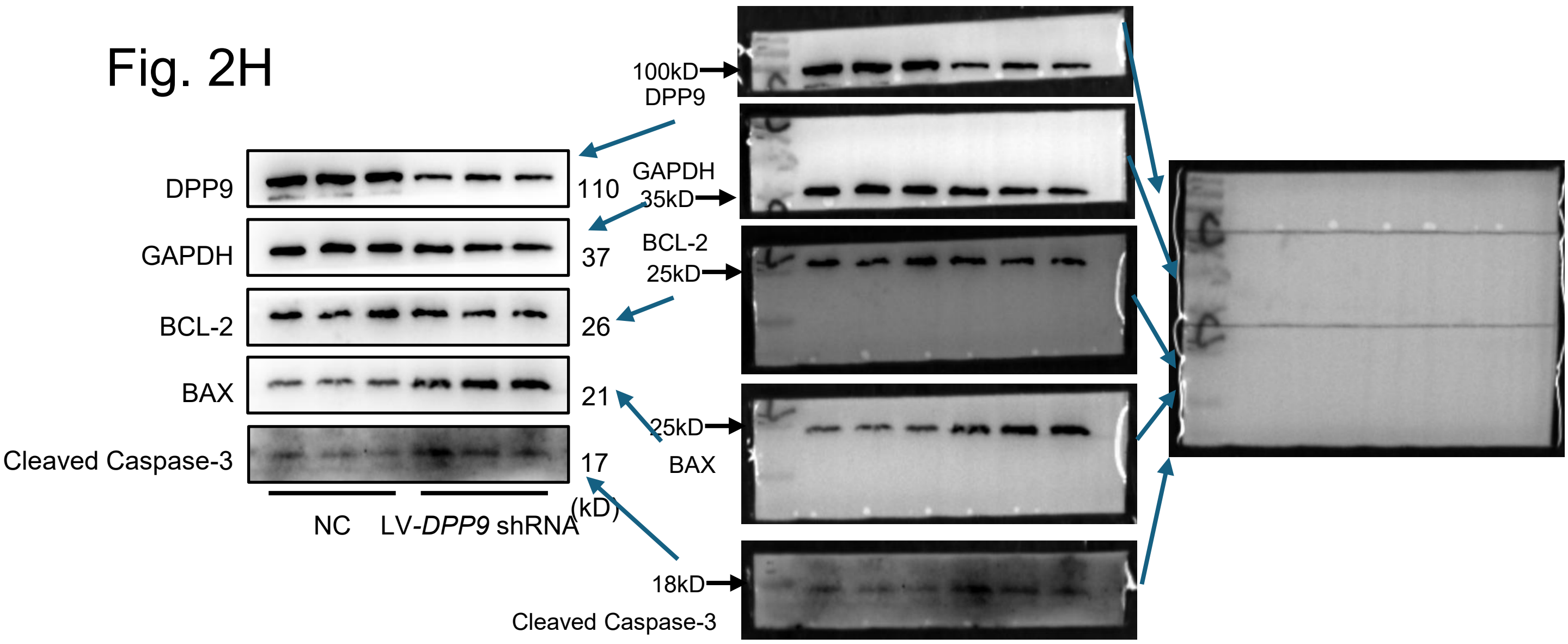

Fig. 2K

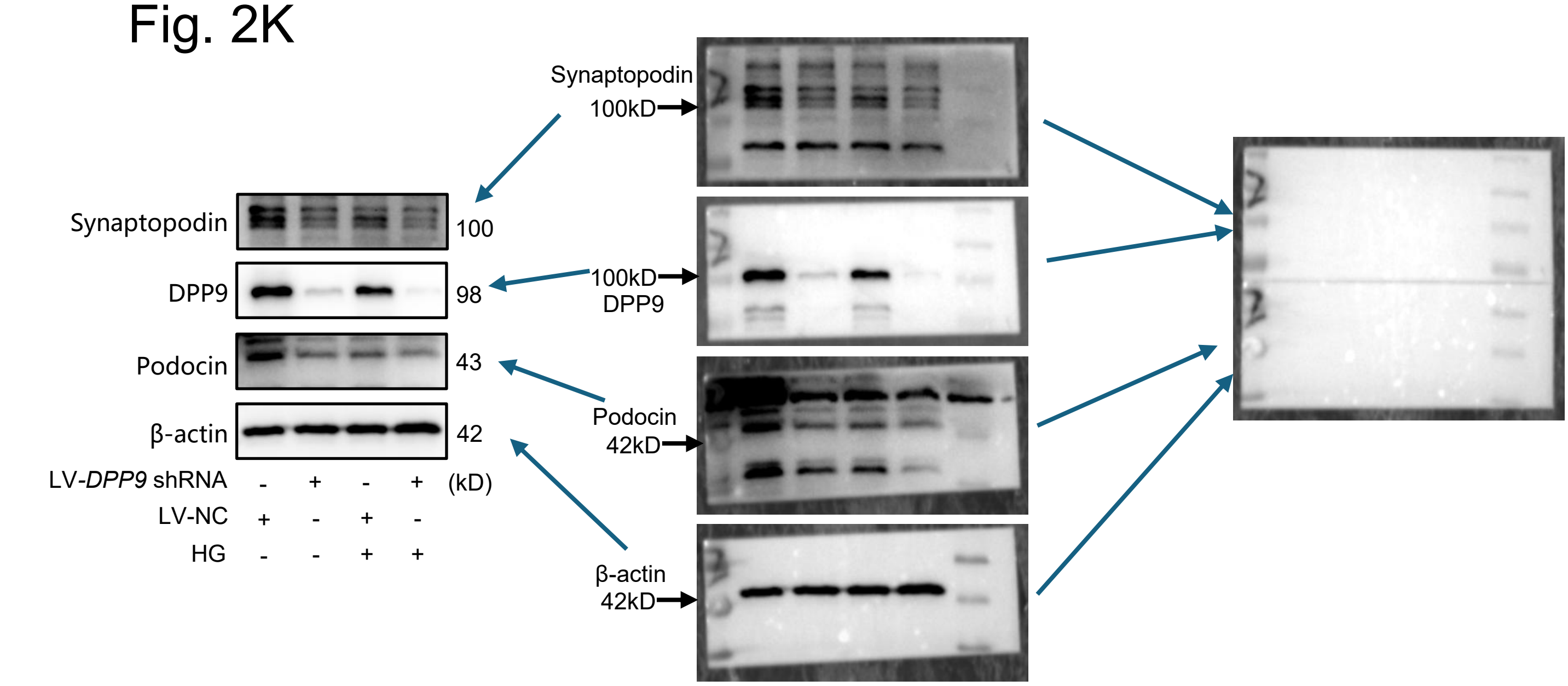

Fig. 2M

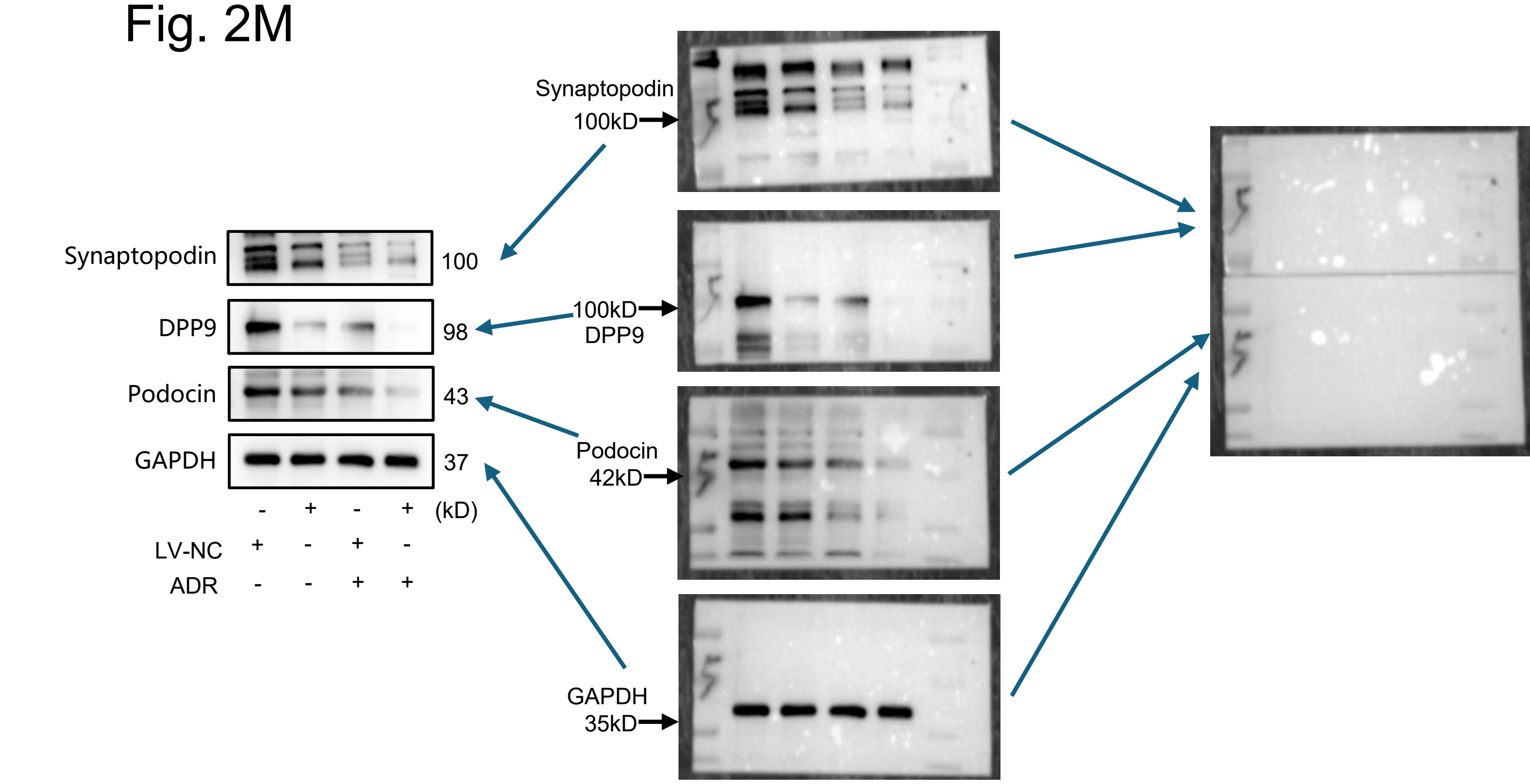

Fig. 3A

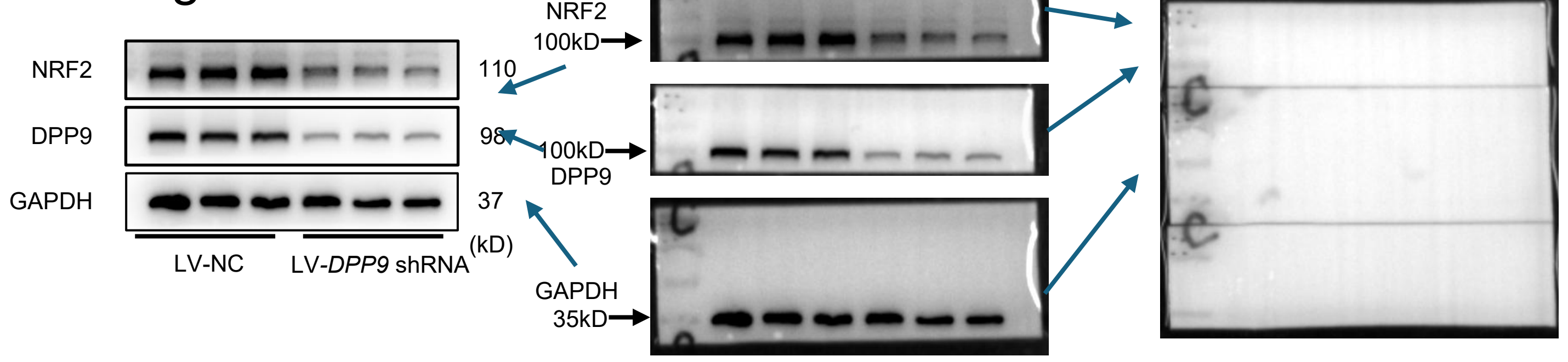

Fig. 3D

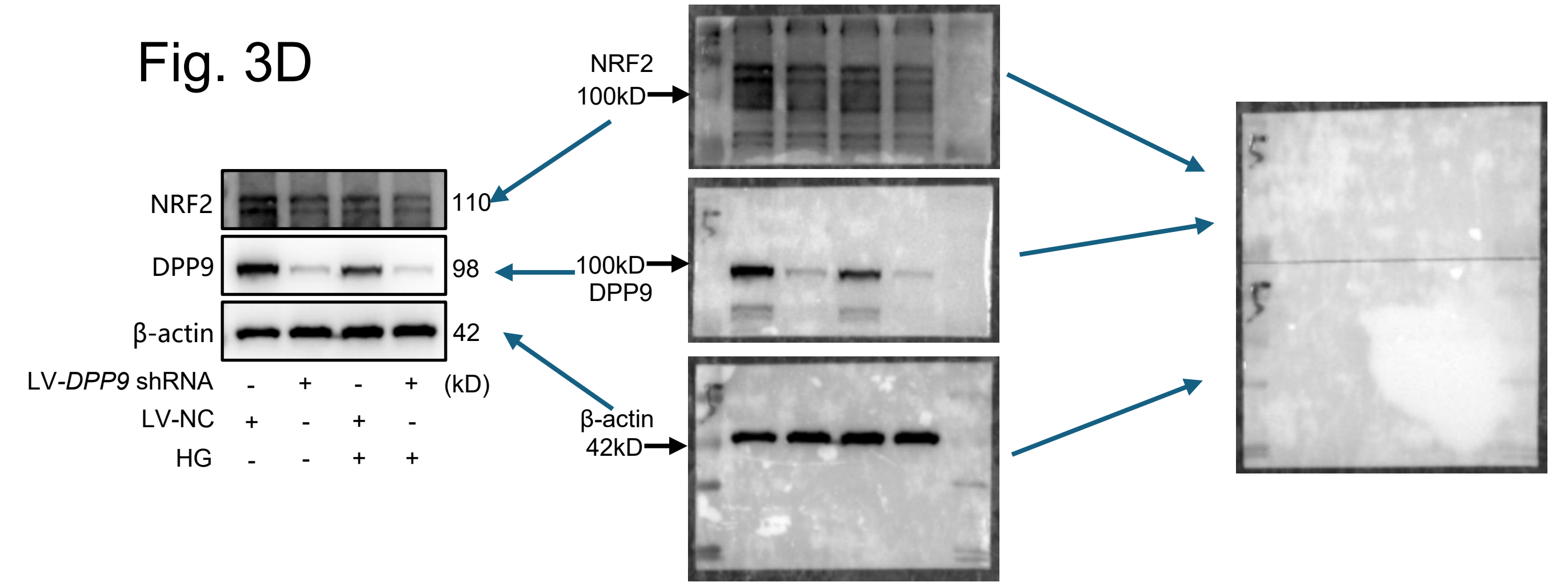

Fig. 3F

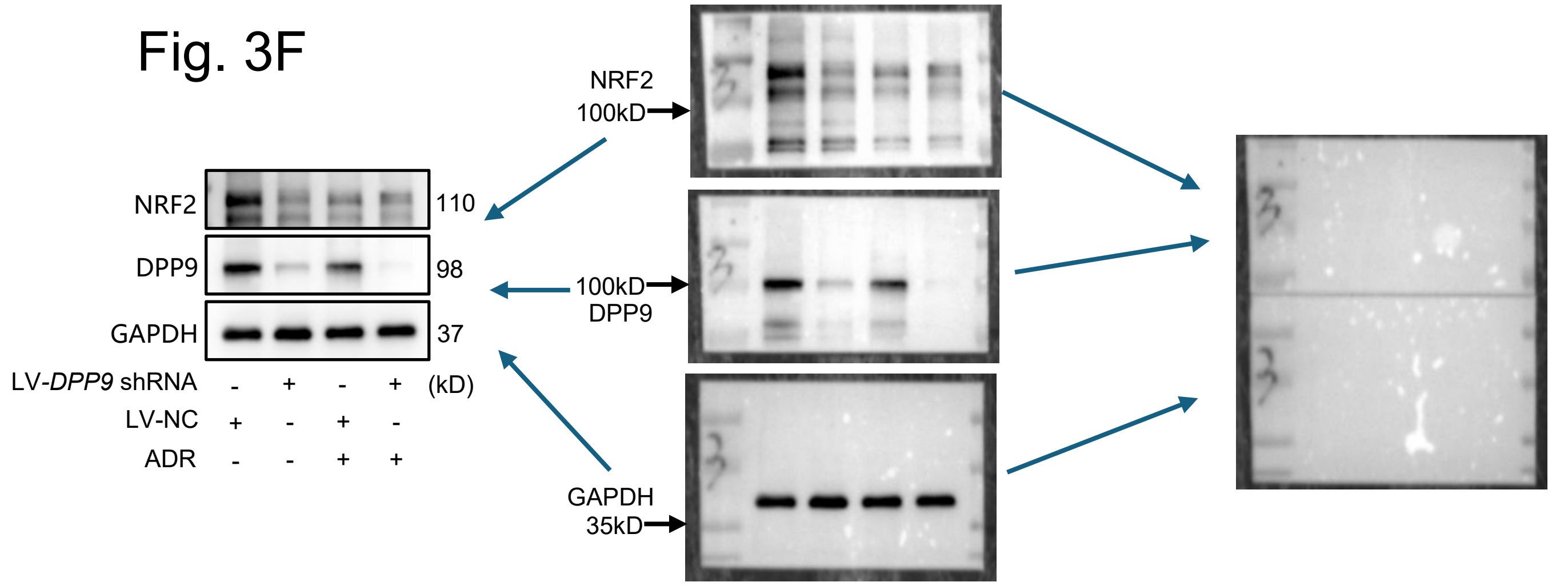

Fig. 3H

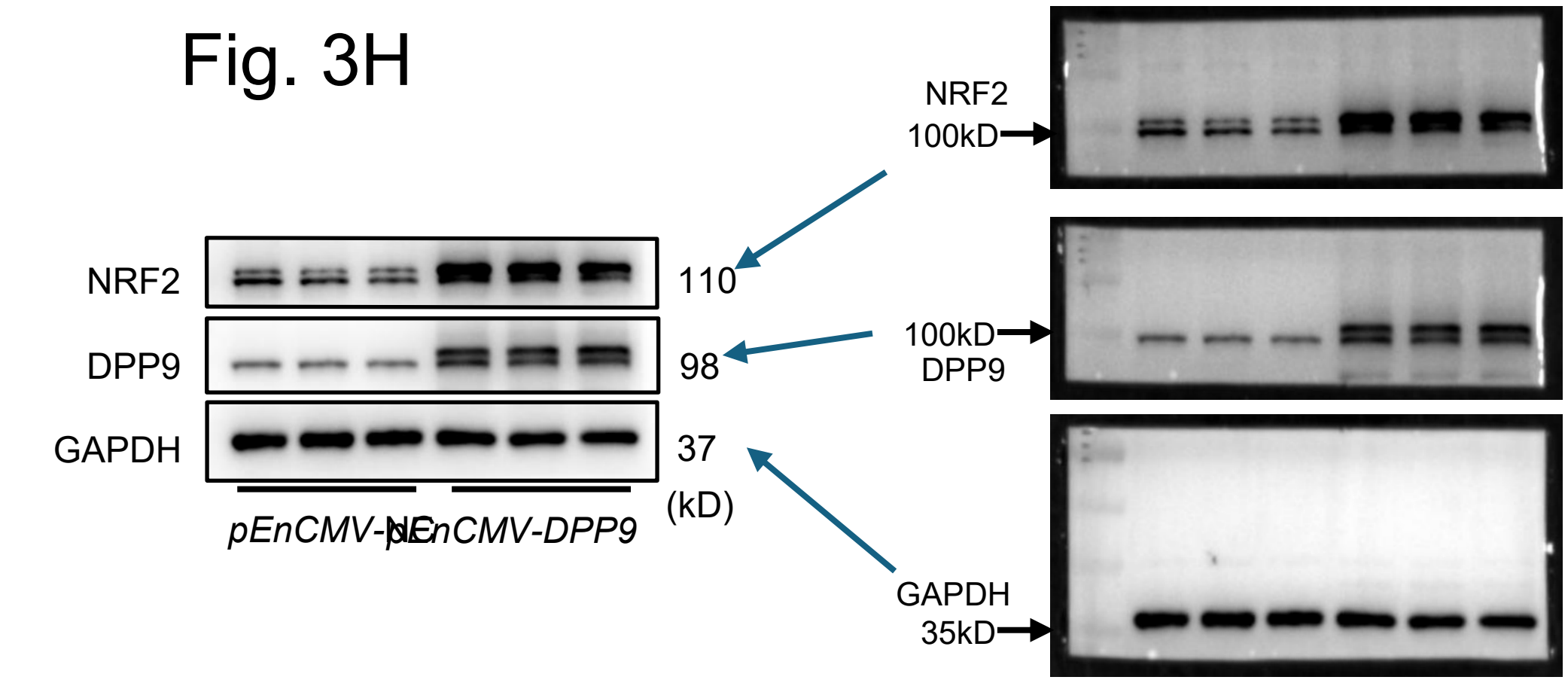

Fig. 3N

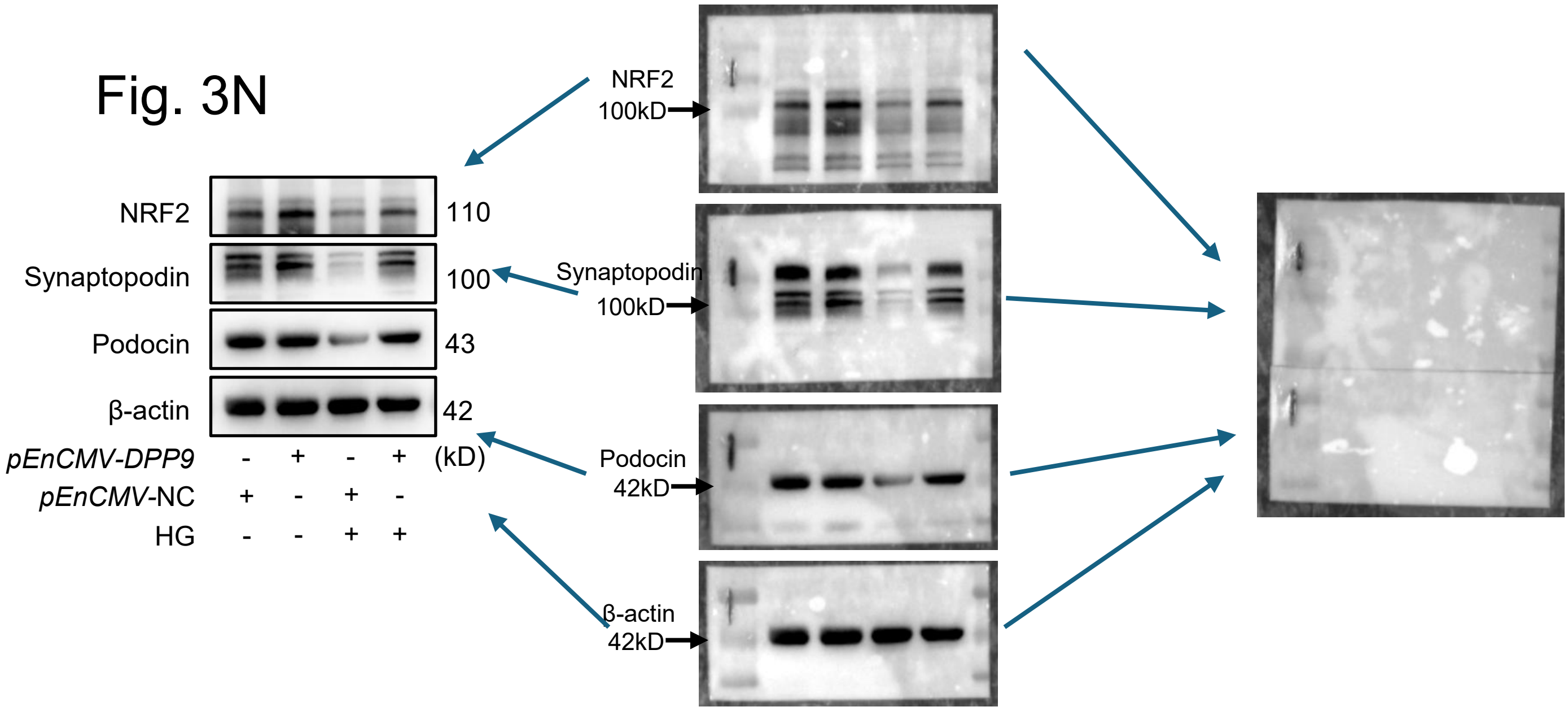

Fig. 3P

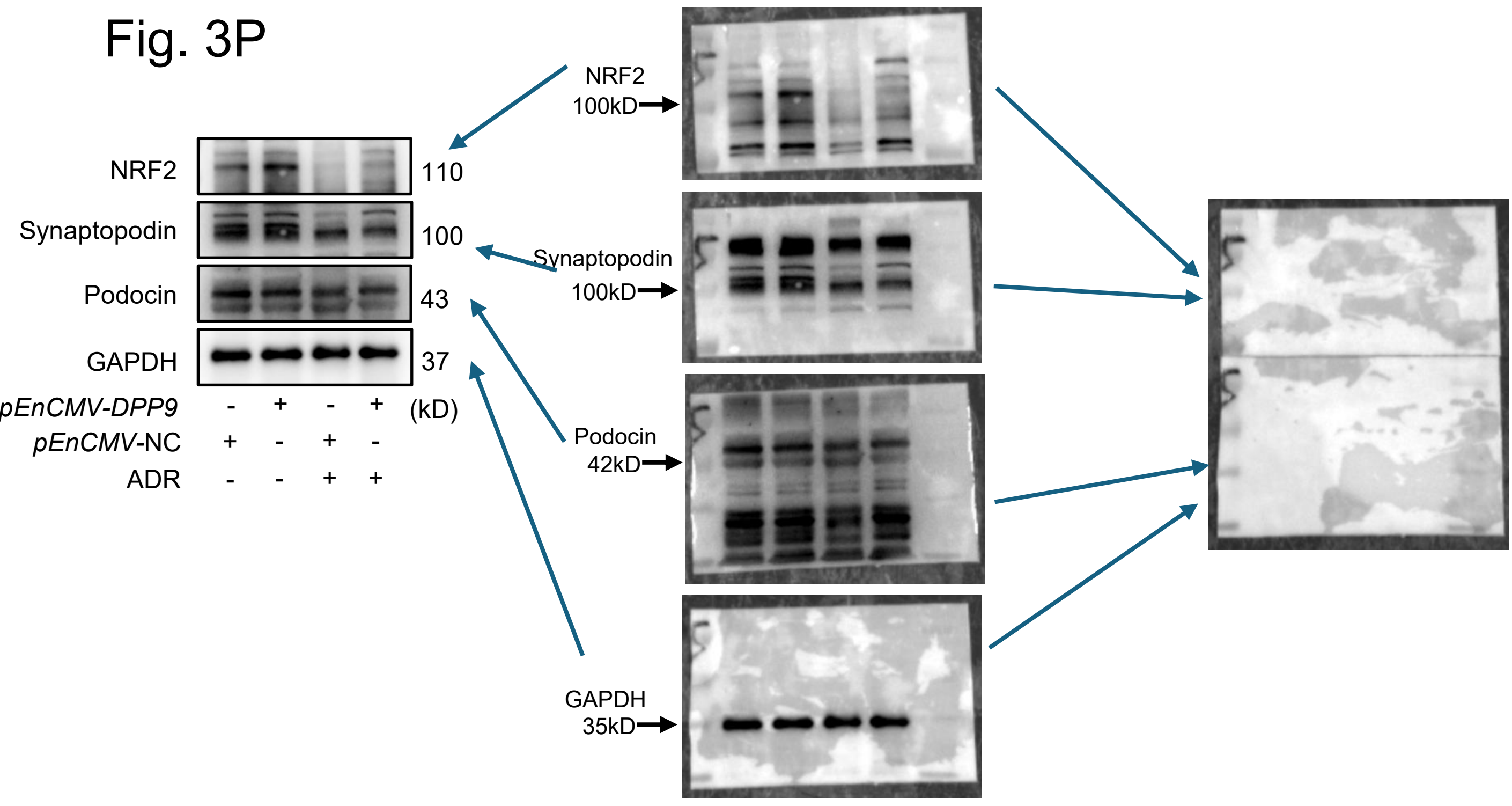

Fig. 5A

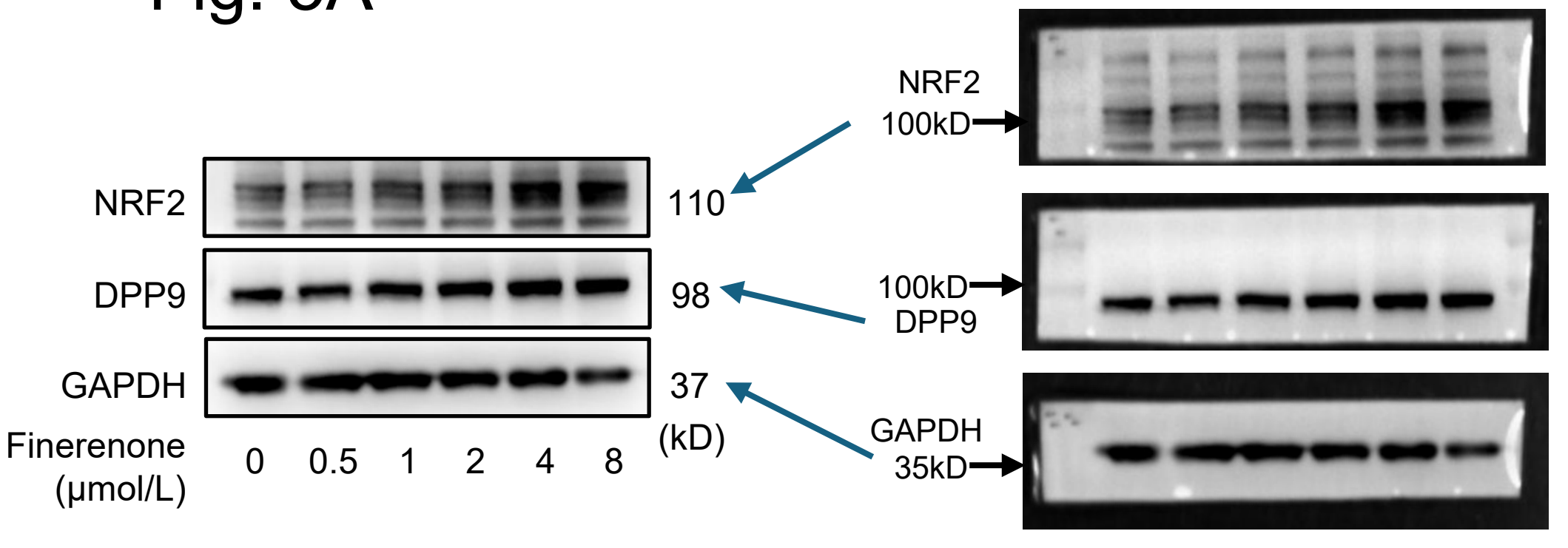

Fig. 5I

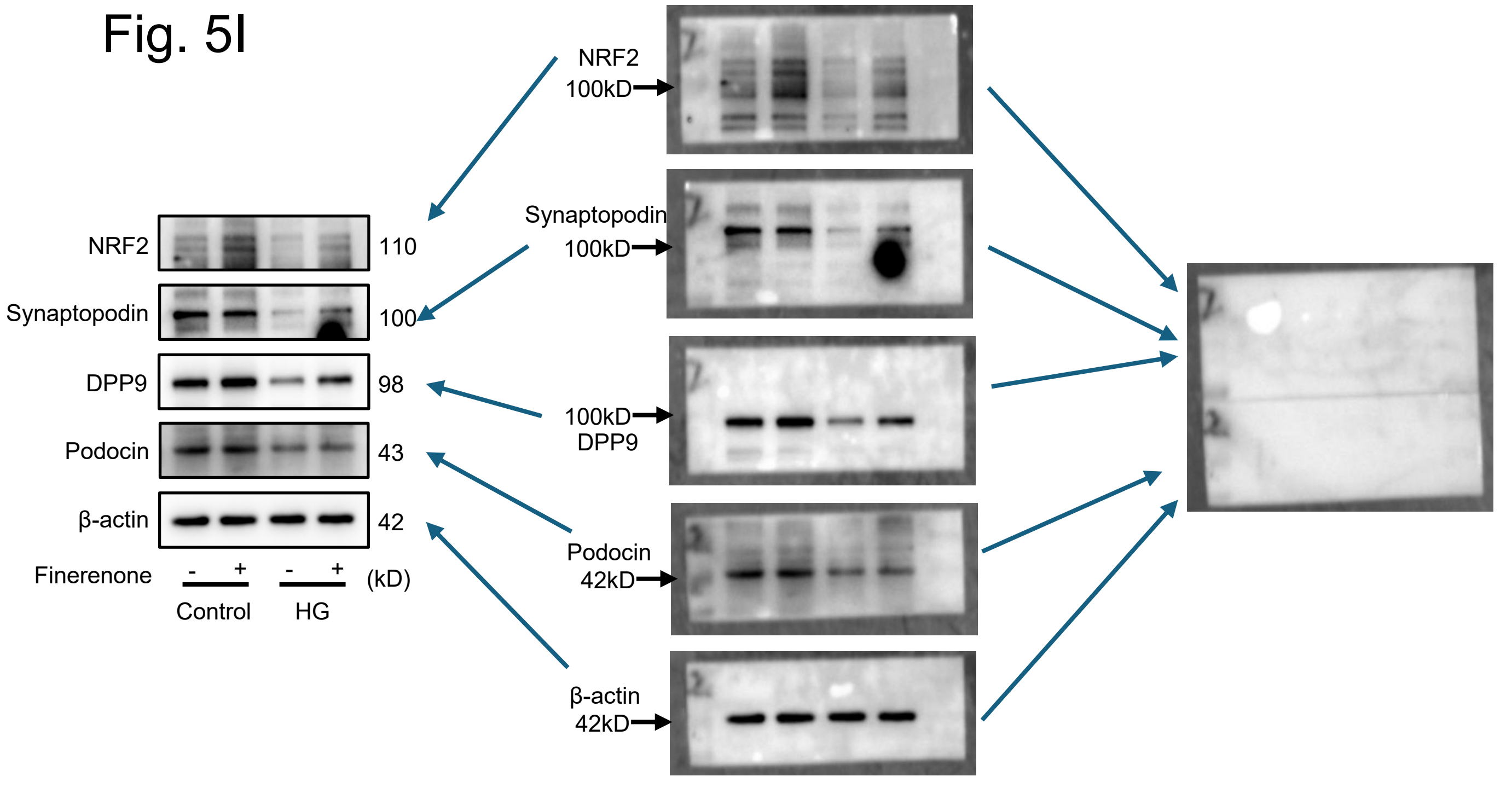

NRF2
Synaptopodin
DPP9
Podocin
β-actin
110
100
98
43
42
Finerenone
- + - +
(kD)
Control
HG
NRF2
100kD
Synaptopodin
100kD
100kD
DPP9
Podocin
42kD
β-actin
42kD

Fig. 5K

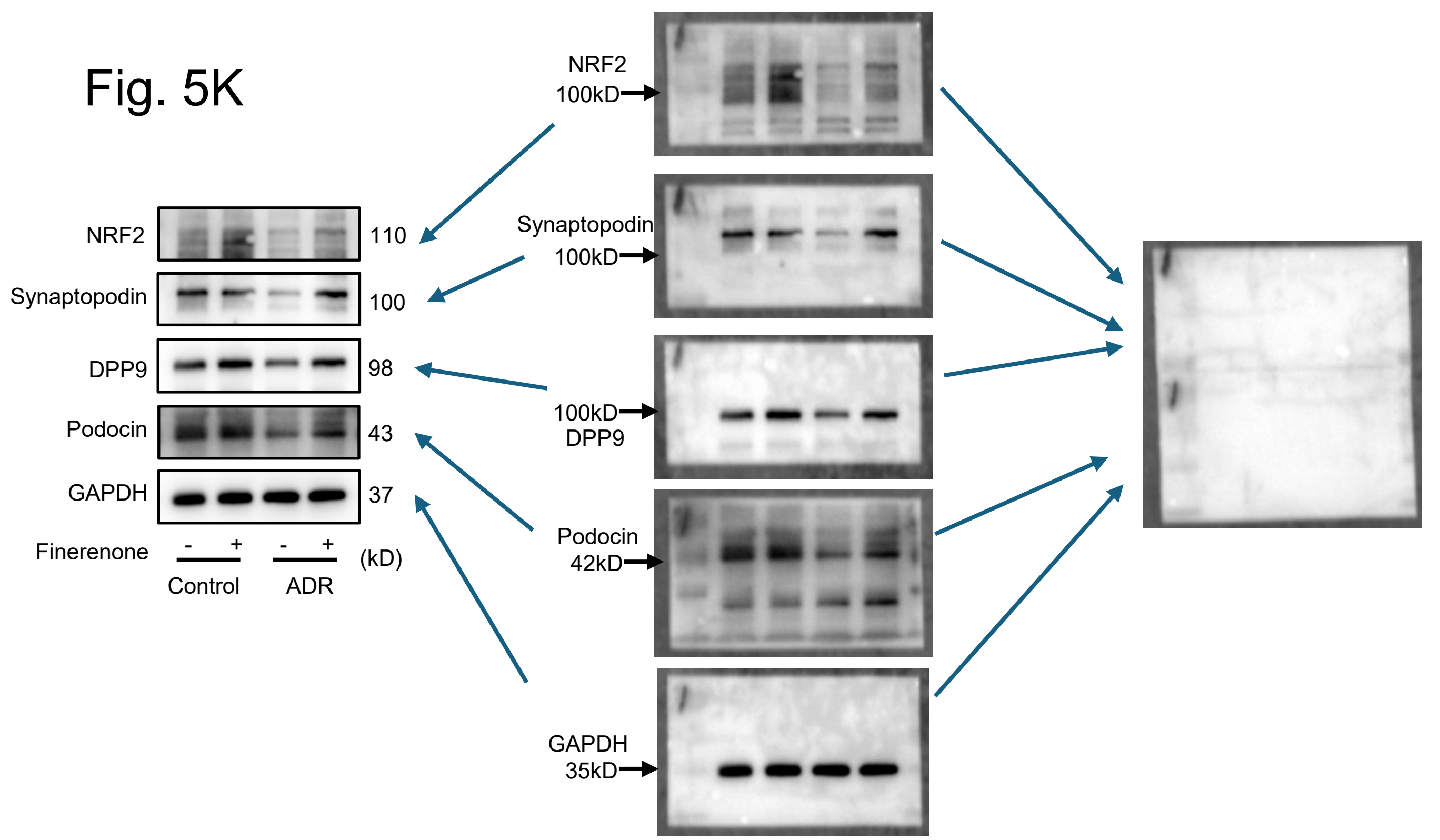

NRF2
Synaptopodin
DPP9
Podocin
GAPDH
110
100
98
43
37
Finerenone
- + - +
(kD)
Control
ADR
NRF2
100kD
Synaptopodin
100kD
100kD
DPP9
Podocin
42kD
GAPDH
35kD

Fig. 6A

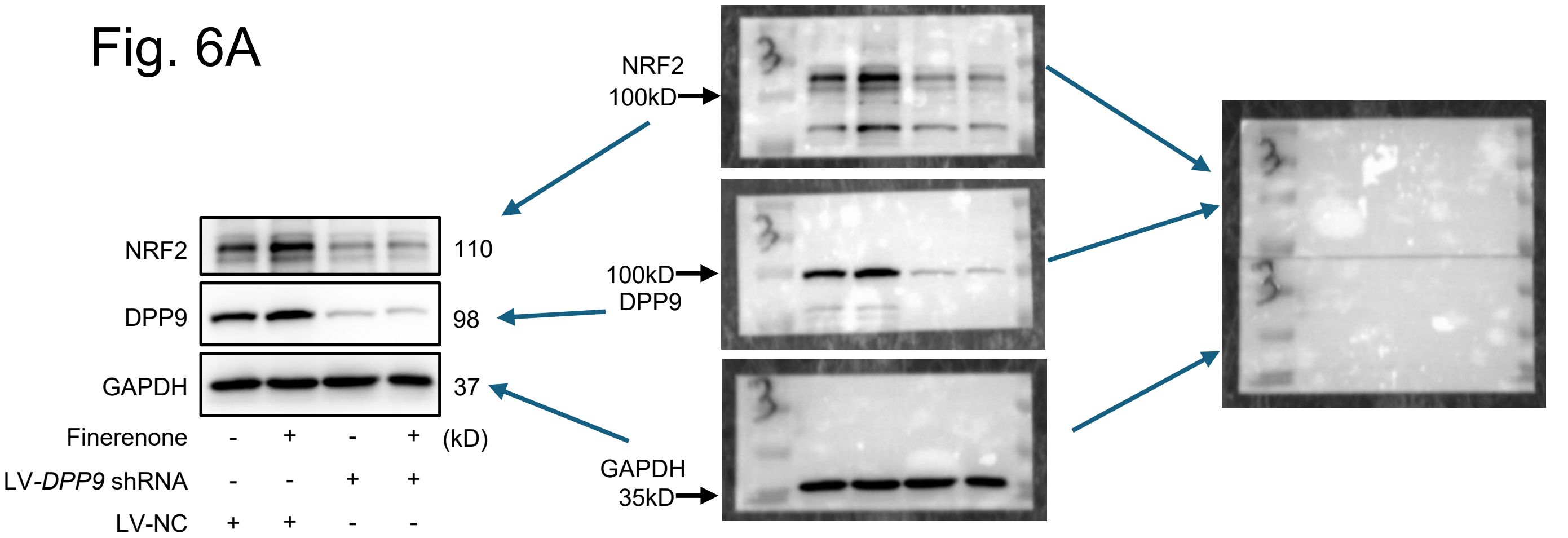
NRF2
100kD
100kD
DPP9
GAPDH
35kD
NRF2
DPP9
GAPDH
110
98
37
Finerenone - + - + (kD)
LV-DPP9 shRNA - - + +
LV-NC + + - -

Fig. 6E

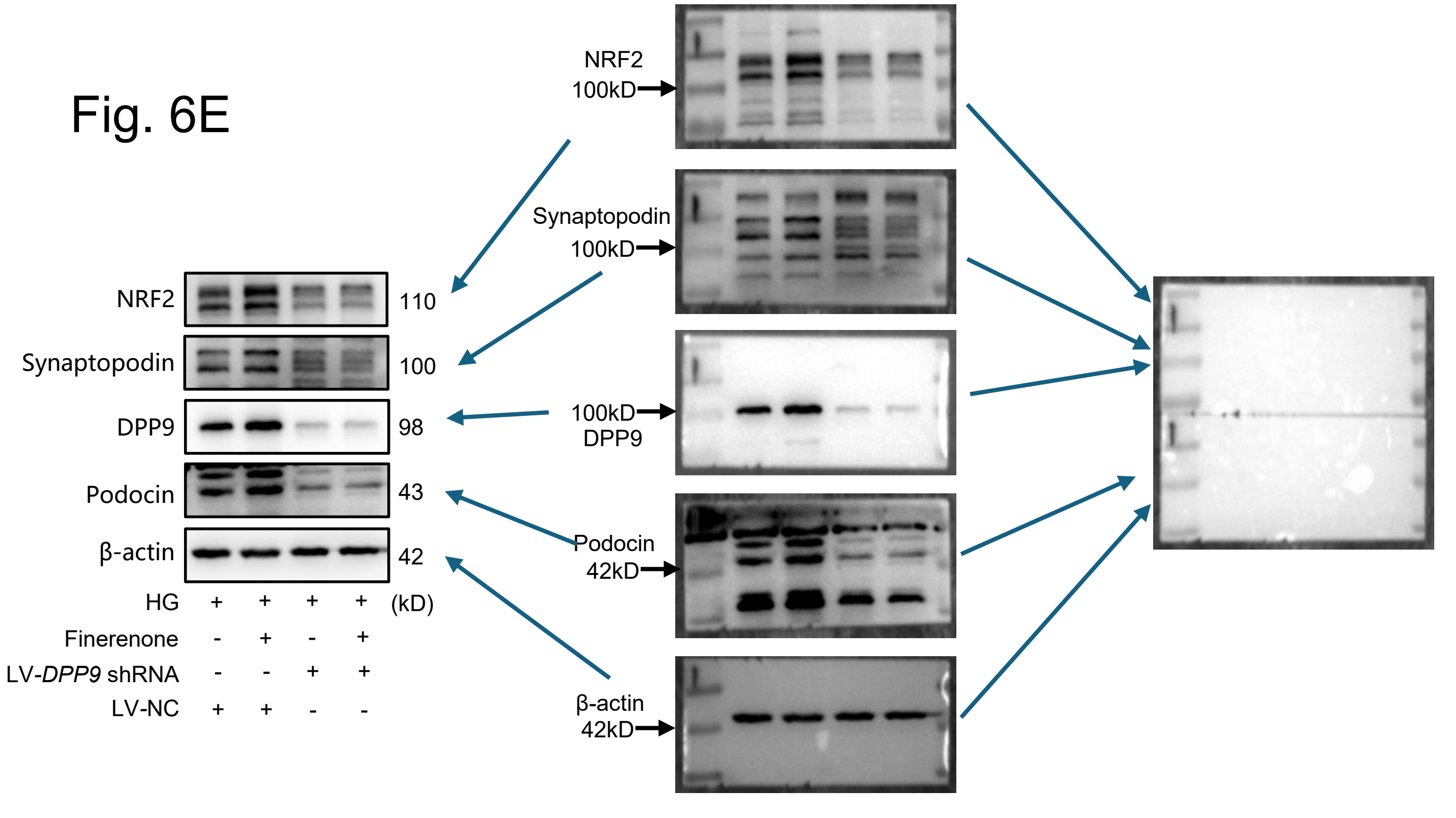
NRF2
100kD
Synaptopodin
100kD
100kD
DPP9
Podocin
42kD
β-actin
42kD
NRF2
Synaptopodin
DPP9
Podocin
β-actin
110
100
98
43
42
HG + + + + (kD)
Finerenone - + - +
LV-DPP9 shRNA - - + +
LV-NC + + - -

Fig. 6G

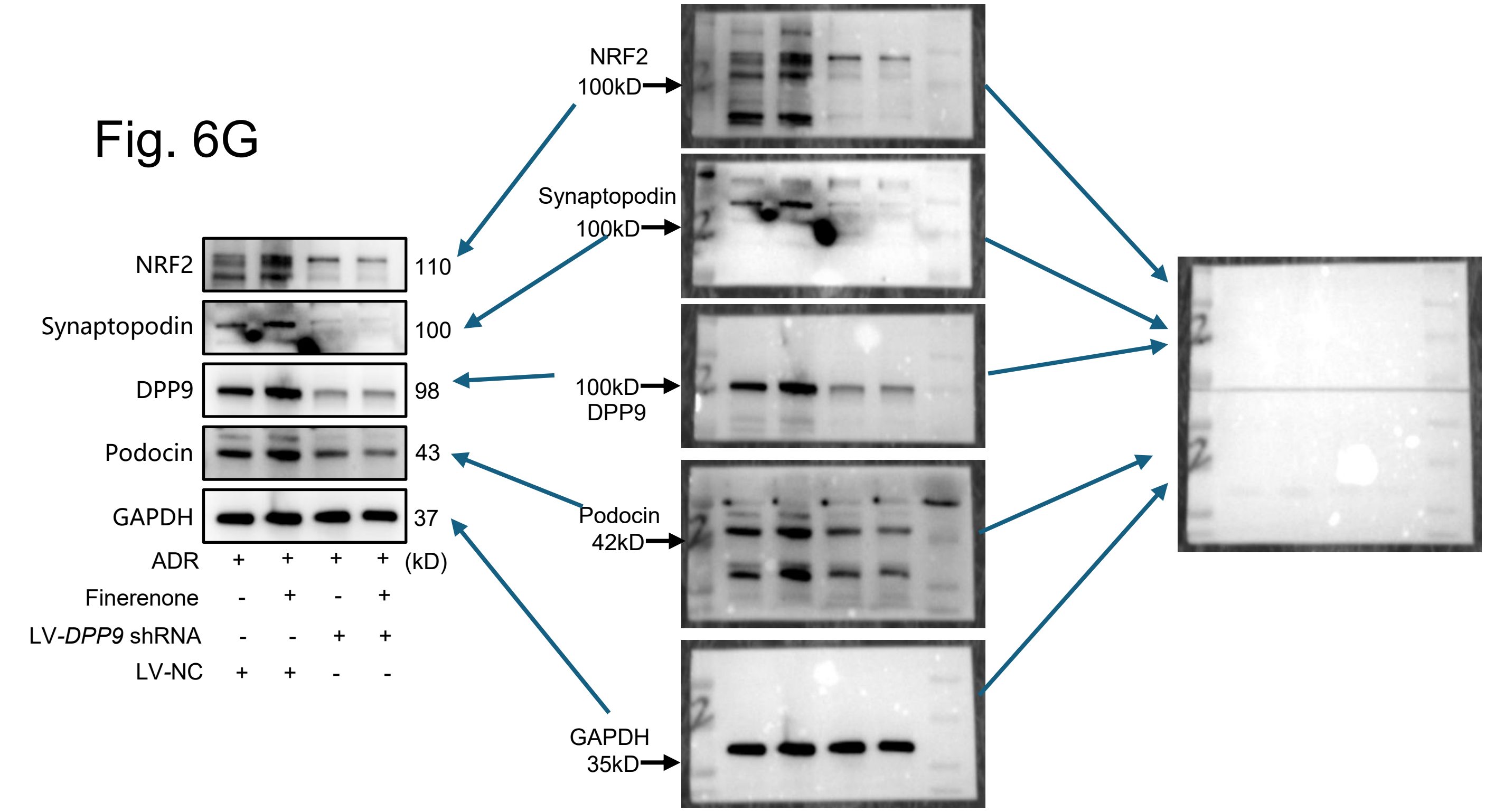

NRF2
Synaptopodin
DPP9
Podocin
GAPDH
110
100
98
43
37
ADR + + + + (kD)
Finerenone - + - +
LV-*DPP9* shRNA - - + +
LV-NC + + - -
NRF2
100kD
Synaptopodin
100kD
100kD
DPP9
Podocin
42kD
GAPDH
35kD

Fig. 6I

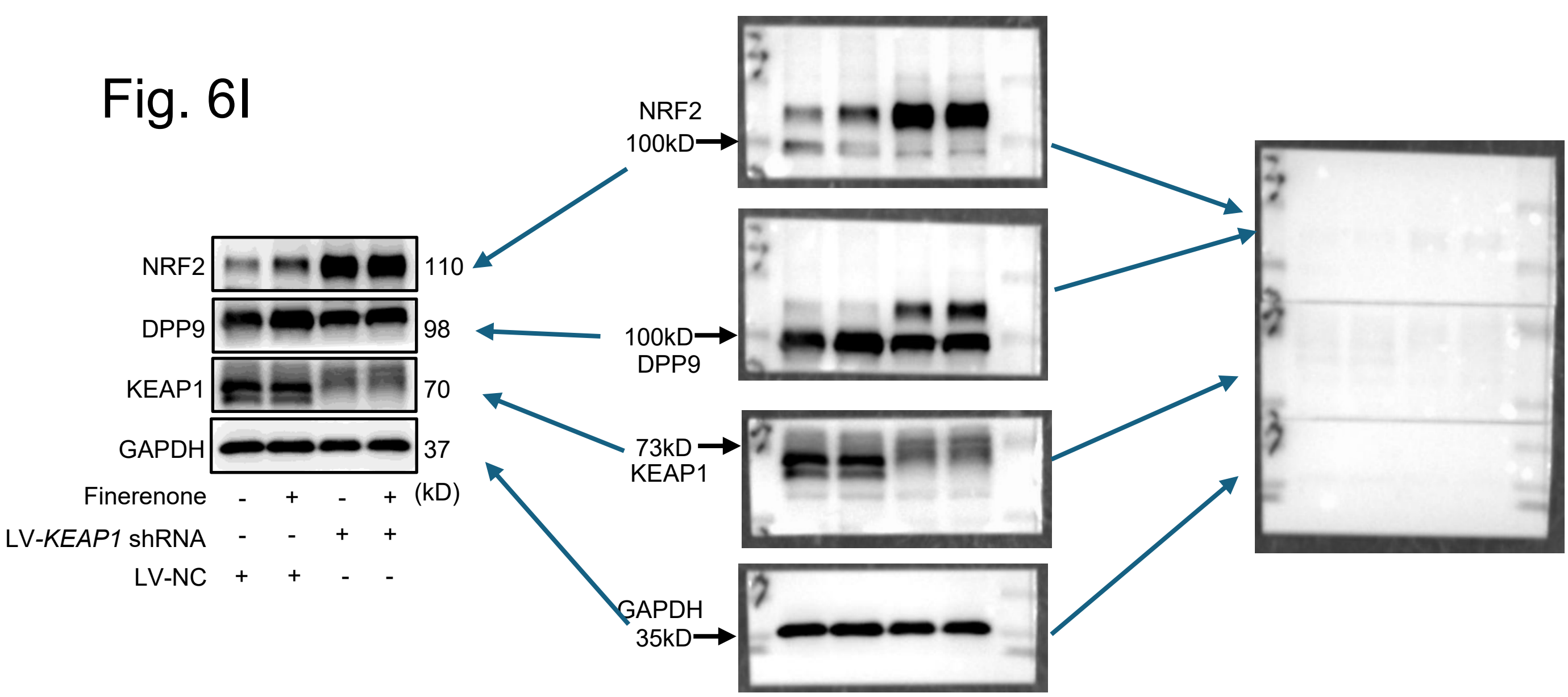

NRF2
DPP9
KEAP1
GAPDH
110
98
70
37
Finerenone - + - + (kD)
LV-*KEAP1* shRNA - - + +
LV-NC + + - -
NRF2
100kD
100kD
DPP9
73kD
KEAP1
GAPDH
35kD

Fig. 6K

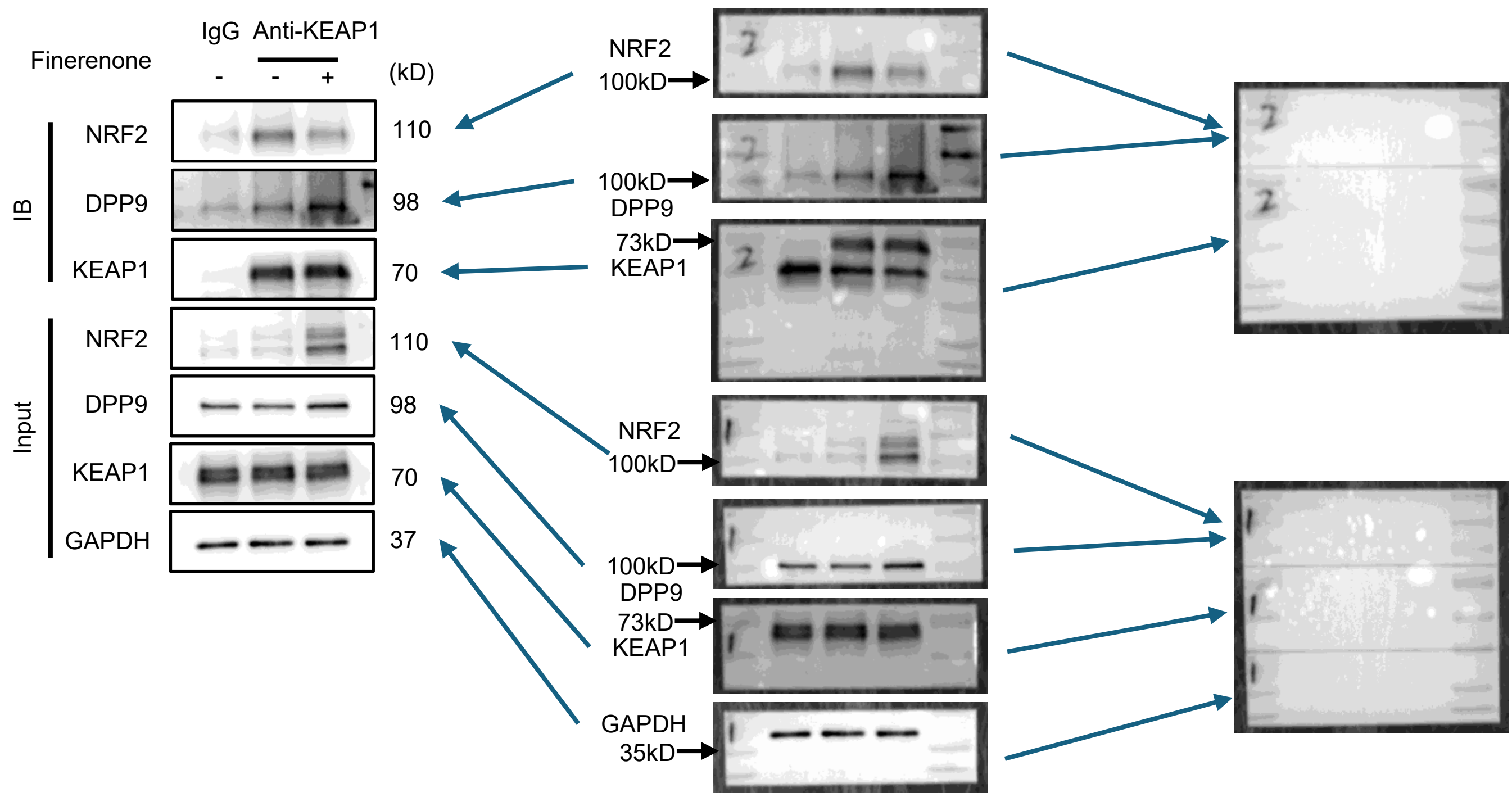
IgG Anti-KEAP1
Finerenone
- - +
(kD)
IB
NRF2 110
DPP9 98
KEAP1 70
Input
NRF2 110
DPP9 98
KEAP1 70
GAPDH 37
NRF2
100kD
100kD
DPP9
73kD
KEAP1
NRF2
100kD
100kD
DPP9
73kD
KEAP1
GAPDH
35kD

Fig. 6M

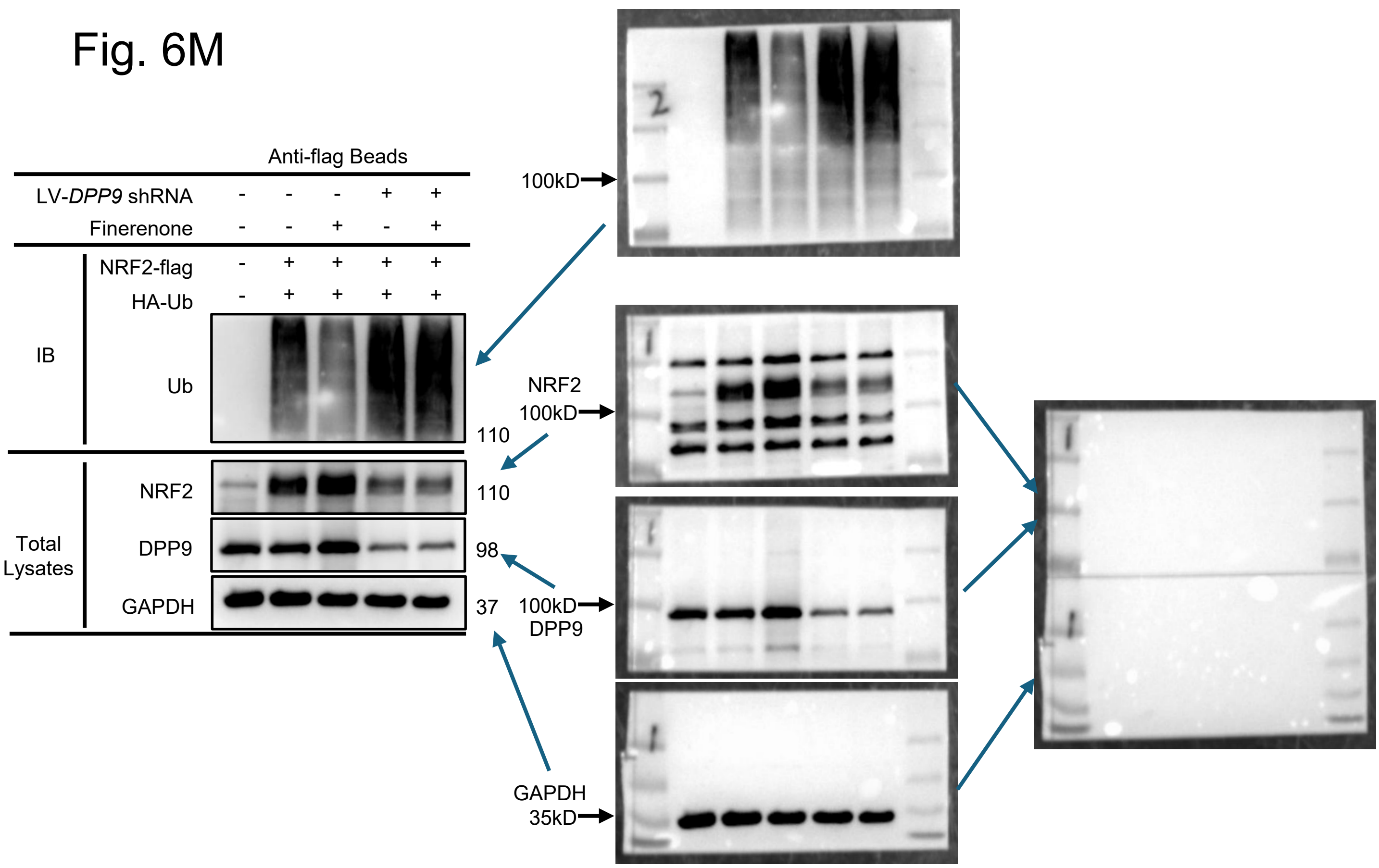
Anti-flag Beads
LV-*DPP9* shRNA - - - + +
Finerenone - - + - +
NRF2-flag - + + + +
HA-Ub - + + + +
IB
Ub
110
Total Lysates
NRF2 110
DPP9 98
GAPDH 37
100kD
NRF2
100kD
100kD
DPP9
GAPDH
35kD

Fig. 7B

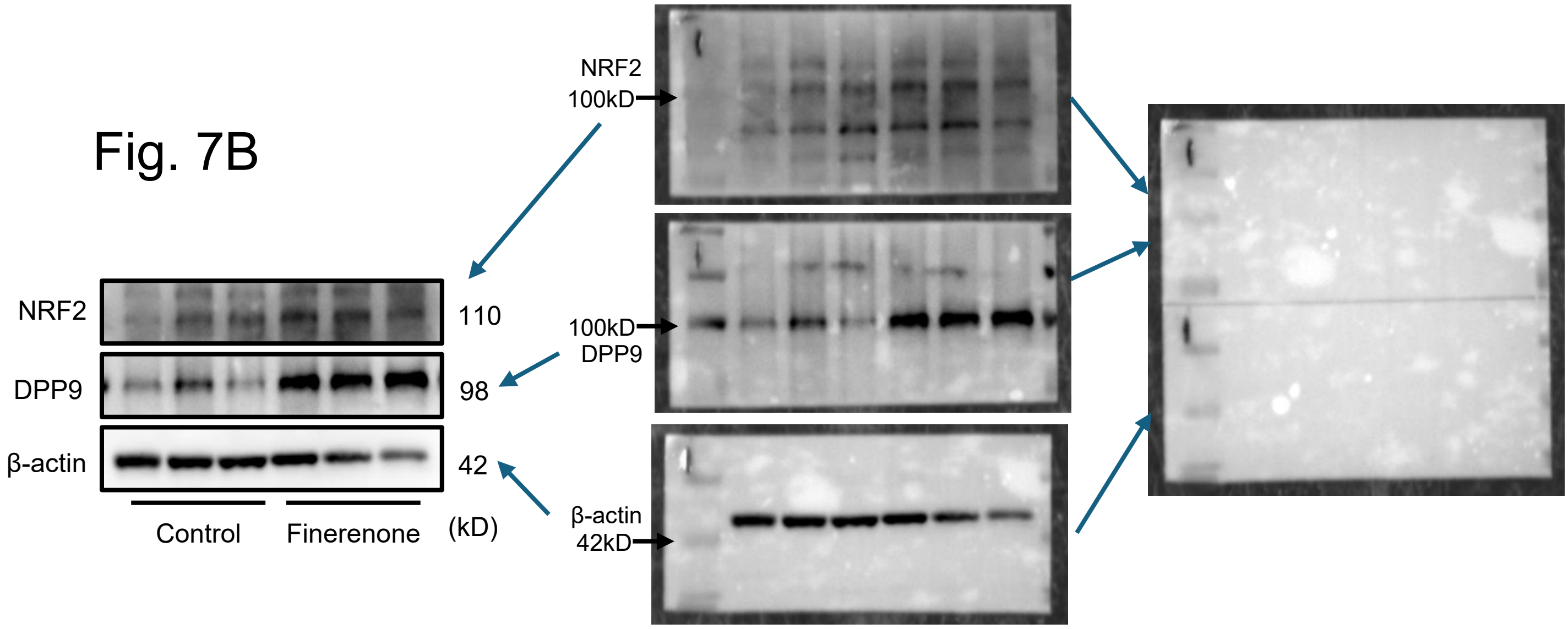

Fig. S2A

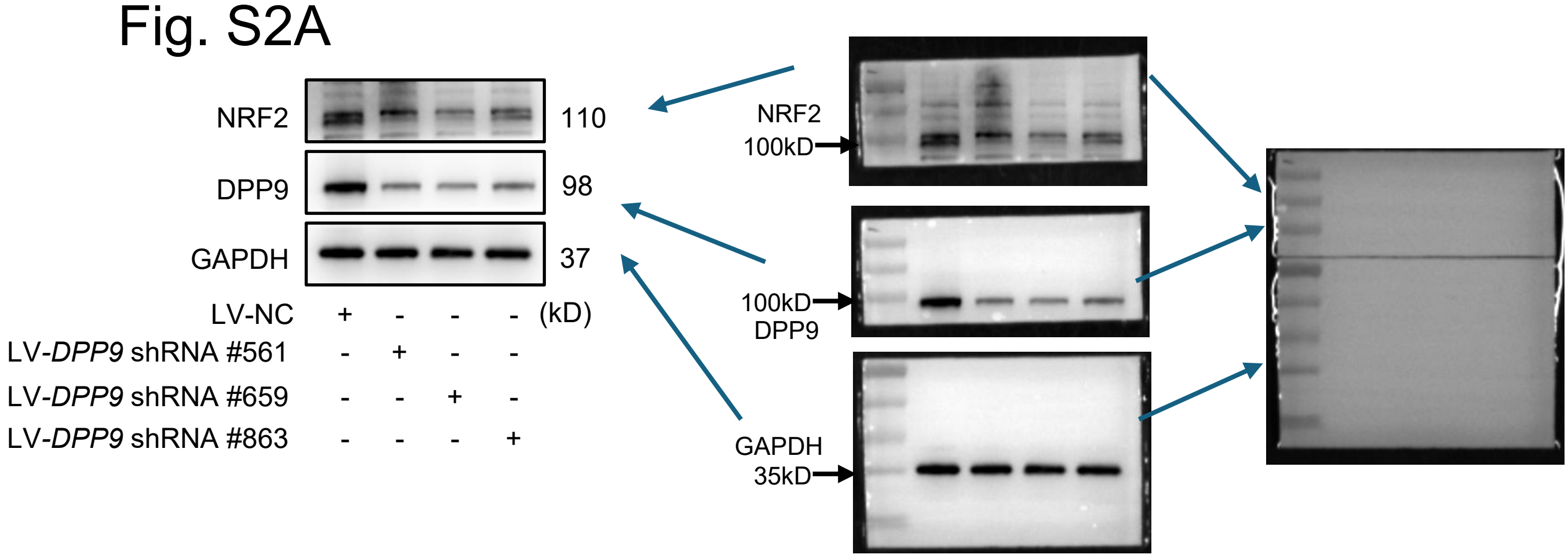

Fig. S3B

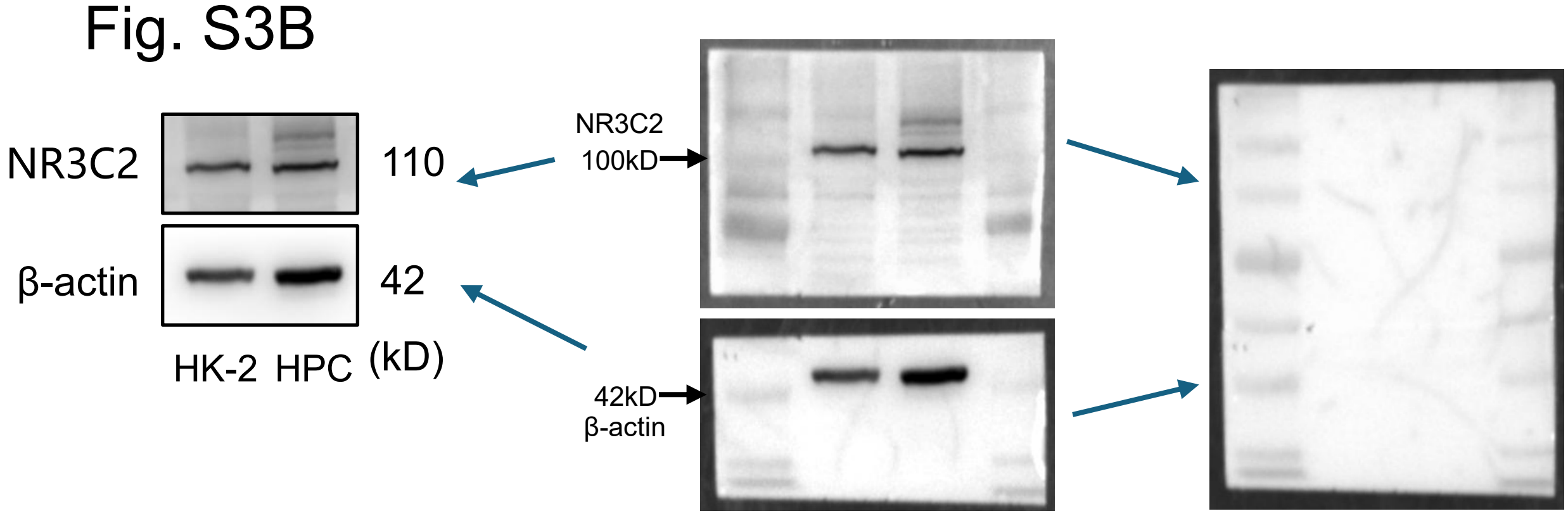

Supplement: Supplementary file 2 [file DataSheet2.PDF]
