# Supplementary material for: Targeting DPP9 attenuates podocyte injury by regulating NRF2 antioxidant signaling
Source: Front Pharmacol. 2026 Sep 17;17:1930044. doi: 10.3389/fphar.2026.1930044 (PMC13627425; doi:10.3389/fphar.2026.1930044)
Supplement: Supplementary file 3 [file Table1.DOCX]

| **Supplementary Table 1. Reagents and Chemicals used in this study** | | |
| --- | --- | --- |
| **Reagents/Chemicals** | **Source** | **Identifier** |
| Streptozocin | Aladdin | Cat# S110910 |
| Adriamycin | Aladdin | Cat# D107159 |
| D- (+) Glucose | Sigma-Aldrich | Cat# G8769 |
| Finerenone | MCE | Cat# HY-111372 |
| PrimeScript RT Master Mix | Takara | Cat# RR036A |
| TB Green Premix Ex Taq | Takara | Cat# RR820A |
| Lipofectamine 3000 transfection reagent | Invitrogen | Cat# L3000 |
| Reactive Oxygen Species Assay Kit | Beyotime Biotechnology | Cat# S0033S |
| Cell Counting Kit-8 | NCM Biotech | Cat# C6005 |
| Western and IP lysis buffer | Beyotime Biotechnology | Cat# P0013 |
| BCA Protein Assay Kit | Beyotime Biotechnology | Cat# P0010 |
| Protein A+G Agarose | Beyotime Biotechnology | Cat# P2055 |
| Anti-Flag Affinity Gel | Beyotime Biotechnology | Cat# P2282 |
| Creatinine assay kit | Nanjing Jiancheng Bioengineering Institute | Cat# C011-2-1 |
| Mouse Albumin ELISA Kit | Proteintech | Cat# KE10117 |
