# Supplementary material for: Targeting DPP9 attenuates podocyte injury by regulating NRF2 antioxidant signaling
Source: Front Pharmacol. 2026 Sep 17;17:1930044. doi: 10.3389/fphar.2026.1930044 (PMC13627425; doi:10.3389/fphar.2026.1930044)
Supplement: Supplementary file 4 [file Table2.DOCX]

| **Supplementary Table 2. Antibodies used in this study** | | |
| --- | --- | --- |
| **Antibodies** | **Source** | **Identifier** |
| Rabbit Polyclonal DPP9 antibody | Abcam | Cat# ab42080 |
| Rabbit Monoclonal DPP9 antibody | Abcam | Cat# ab302903 |
| Rabbit Polyclonal DPP9 antibody | Affinity | Cat# DF13332 |
| Guinea Pig polyclonal Synaptopodin antibody | Origene | Cat# AP33487SU-N |
| Mouse Monoclonal Synaptopodin antibody | Santa Cruz Biotechnology | Cat# sc-515842 |
| Rabbit Polyclonal NRF2 antibody | Proteintech | Cat# 16396-1-AP |
| Rabbit Polyclonal KEAP1 antibody | Proteintech | Cat# 10503-2-AP |
| Rabbit Polyclonal Podocin antibody | Sigma-Aldrich | Cat# P0372 |
| Rabbit Polyclonal BCL-2 antibody | Wanleibio | Cat# WL01556 |
| Rabbit Polyclonal BAX antibody | Wanleibio | Cat# WL01637 |
| Rabbit Polyclonal Cleaved Caspase-3 antibody | Wanleibio | Cat# WL01992 |
| Rabbit Polyclonal NR3C2 antibody | Proteintech | Cat# 21854-1-AP |
| Rabbit Polyclonal Ubiquitin antibody | Proteintech | Cat# 10201-2-AP |
| Rabbit Monoclonal Wilms Tumor Protein antibody | Abcam | Cat# ab89901 |
| Mouse Monoclonal GAPDH antibody | Proteintech | Cat# 60004-1-Ig |
| Mouse Monoclonal Beta Actin antibody | Proteintech | Cat# 66009-1-Ig |
| HRP-conjugated Goat Anti-Mouse IgG(H+L) | Proteintech | Cat# SA00001-1 |
| HRP-conjugated Goat Anti-Rabbit IgG(H+L) | Proteintech | Cat# SA00001-2 |
| Donkey Anti-Rabbit IgG (H+L) (Alexa Fluor® 488) | Jackson ImmunoResearch | Cat# 711-545-152 |
| Donkey Anti-Mouse IgG (H+L) (Alexa Fluor® 594) | Jackson ImmunoResearch | Cat# 715-585-151 |
| Donkey Anti-Guinea Pig IgG (H+L) (Alexa Fluor® 594) | Jackson ImmunoResearch | Cat# 706-585-148 |
