# Supplementary material for: Targeting DPP9 attenuates podocyte injury by regulating NRF2 antioxidant signaling
Source: Front Pharmacol. 2026 Sep 17;17:1930044. doi: 10.3389/fphar.2026.1930044 (PMC13627425; doi:10.3389/fphar.2026.1930044)
Supplement: Supplementary file 5 [file Table3.DOCX]

| **Supplementary Table 3. Primers for Quantitative real-time PCR** | | |
| --- | --- | --- |
| **Species** | **Gene** | **Sequence** |
| Human | DPP9 | sense 5’-ACACTGAGCGCTACATGGAC-3’ antisense 5’-CAAGCGGTTGGGCTCATTG-3’ |
| Human | HMOX-1 | sense 5’-CTTTCAGAAGGGCCAGGTGA -3’ antisense 5’-GTAGACAGGGGCGAAGACTG-3’ |
| Human | GCLM | sense 5’-TCCCATGCAGTGGAGAAGAT-3’ antisense 5’-AGCTGTGCAACTCCAAGGAC-3’ |
| Human | AKR1C1 | sense 5’-GGAGGCCATGGAGAAGTGTA-3’ antisense 5’-GCACACAGGCTTGTACCTGA-3’ |
